# Allosteric DNAzyme‐Enabled Sensitive and Multiplex Detection of Biomarkers for Rapid Diagnosis of Urinary Tract Infections

**DOI:** 10.1002/advs.75417

**Published:** 2026-04-20

**Authors:** Yanzhe Shen, Chenzhi Shi, Xiaowei Ma, Pengfei Hou, Haomin Zhang, Juanxiu Qin, Li Pan, Guilin Li, Lifei Gao, Qian Ma, Donglei Yang, Min Li, Pengfei Wang

**Affiliations:** ^1^ Institute of Molecular Medicine Department of Laboratory Medicine Shanghai Key Laboratory for Nucleic Acid Chemistry and Nanomedicine Renji Hospital School of Medicine Shanghai Jiao Tong University Shanghai China; ^2^ National Eco & Tech Zone Autobio Diagnostics Co., Ltd. Zhengzhou Henan China; ^3^ Intellinosis Biotechnology Co., Ltd. Shanghai China

**Keywords:** biomarkers, clinical diagnosis, DNAzyme, multiplex detection, urinary tract infection

## Abstract

Urinary tract infections (UTI) are among the most prevalent infectious diseases, demanding rapid diagnosis to provide timely therapeutic interventions. Urinary molecules can indicate the responses of the host cells to pathogenic infections, which may serve as markers for UTI diagnosis. Sensitive and multiplex detection of urinary markers in one‐pot has remained challenging. Herein, we developed an allosteric DNAzyme‐based biosensor, denoted as SMART (sensitive and multiplex detection of ATP and miRNAs for UTI diagnosis), to enable rapid UTI diagnosis. Multiple sensing modules were integrated into a unimolecular DNA strand of perfect stoichiometry and excellent thermodynamic stability that collectively led to enhanced detection capability. The binding of targets to the detection module induces conformational reconfiguration of SMART to activate sequence‐specific catalytic cleavage against fluorescent RNA reporters. SMART demonstrated remarkable sensitivity (ATP: ∼pM; miRNAs: ∼fM) and multiplexing capability (∼4 markers) to realize extraction‐free, preamplification‐free, and rapid (∼2.5 hr) detection of UTI markers. SMART‐based UTI diagnosis model yielded a detection accuracy of 95.5% in a cohort of 164 patients. SMART may serve as a technical platform for detecting various markers that could be applied to the diagnosis of UTI and many other diseases in the future.

## Graphical abstract

A catalytic allosteric DNAzyme assay (SMART) is developed by engineering DNAzyme into a unimolecular biosensor, enabling highly sensitive and multiplex detection of small molecules and nucleic acids. SMART enables extraction‐free, one‐pot, one‐step, isothermal, and cost‐effective detection of biological markers, achieving >95% accuracy for rapid urinary tract infection diagnosis and offering a versatile platform for molecular disease diagnostics.

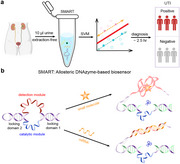

## 1 Introduction

Urinary tract infections (UTI) are pathogenic microbial invasions (e.g., bacteria, fungi, or viruses) to the urinary system, ranking among the most prevalent infectious diseases [1, 2]. Clinical diagnosis of UTI relies on the gold‐standard urine culture method via cultivation of clean‐catch midstream urine samples, which suffers from prolonged turnaround time (24‐48 h), limited sensitivity, and may fail to detect infections caused by fastidious, slow‐growing, or anaerobic pathogens, delaying therapeutic interventions [3, 4, 5]. There is an emerging need to develop sensitive, rapid, and convenient assays for the diagnosis of UTI to enable prompt therapeutic treatment of infected patients. Inflammatory responses of the host cells may induce significant changes to a variety of biological molecules (e.g., ATP, microRNAs, etc.), which have demonstrated large potential as diagnostic markers for UTI [6, 7, 8, 9, 10]. Conventional detection methods for small molecules, such as high‐performance liquid chromatography (HPLC) and mass spectrometry (MS) are hampered by low sensitivity, high cost, complex sample preparation, and lengthy procedures, rendering them unsuitable for sensitive and rapid UTI diagnosis [11, 12, 13]. Newly developed methods predominantly rely on fluorescence or colorimetric readouts, for instance, the luciferin‐luciferase bioluminescence system for ATP detection [14, 15]. Nevertheless, as an enzyme‐dependent platform, it suffers from limitations such as susceptibility to enzymatic instability, environmental interference, and compromised selectivity due to coexisting ions [15, 16]. For microRNA (miRNA) detection, reverse transcription quantitative polymerase chain reaction (RT‐qPCR) remains the gold standard method due to its high sensitivity, but it requires laborious pre‐processing steps (e.g., RNA extraction, reverse transcription), a centralized laboratory, and trained personnel [17, 18]. Besides, it lacks the capability to detect markers other than nucleic acids, rendering it unable to realize simultaneous one‐pot detection of multiple types of markers [19]. Many isothermal nucleic acid amplification assays have been developed for miRNA detection to minimize the need for complicated instruments, including rolling circle amplification (RCA) [20, 21], loop‐mediated isothermal amplification (LAMP) [22, 23], reverse transcription recombinase polymerase amplification (RT‐RPA) [24, 25], and CRISPR‐Cas [26, 27] technologies. Although they have achieved remarkable sensitivity, they are often accompanied by limitations such as nonspecific detection (e.g., off‐target amplification, aerosol contamination), serial operations, and the need for expensive enzymes. There is an emerging need to develop an ultrasensitive and integrated method to omit the need for extraction, preamplification, and protein‐based enzymes for the simultaneous detection of miRNAs and small molecules in one pot.

DNAzyme is a type of in vitro‐selected synthetic DNA molecules possessing enzyme‐like catalytic activities, for instance, the cleavage of nucleic acid phosphodiester bonds [6, 28, 29, 30, 31, 32], which have been extensively used for detecting various targets including metallic ions [33, 34, 35], small molecules [36, 37], proteins [38, 39, 40], bacteria [41, 42], and nucleic acids [43, 44, 45]. In recent years, considerable advances have been achieved in the development of allosteric or self‐locked DNAzyme systems (e.g., CHAzymi, C‐mDz, and CLARISSA, etc.), and have demonstrated significant innovations in signal amplification, highly specific target recognition, and cellular imaging, underscoring the expanding potential of DNAzyme‐based regulatory platforms in biosensing and molecular diagnostics [46, 47, 48, 49, 50]. However, we also note that many existing systems still face several challenges, including reliance on chemical modifications, complex nanostructure assembly, dependence on sample pre‐extraction and amplification steps, as well as limited validation in clinical samples and insufficient evaluation of interference in complex biological matrices. Conventional DNAzyme‐based biosensors for small molecules and miRNAs generally employed multi‐component molecular architectures, mainly including toehold‐mediated and split‐and‐resume strategies that only achieved a moderate sensitivity, typically in the nanomolar to picomolar range (Figure S1) [6, 51, 52, 53, 54, 55, 56, 57]. These multi‐component designs face intrinsic limitations to limit their detection sensitivity, including stringent stoichiometric ratio requirements, susceptibility to kinetic traps, impaired catalytic efficiency, and non‐specific background leakage [58, 59, 60, 61]. These constraints largely impede the system's capability to detect trace amounts of molecular markers, especially in an extraction‐free and one‐pot multiplex manner, restricting their clinical applications for UTI diagnosis. Recent advances in UTI diagnostics and 10–23 DNAzyme biosensing have significantly advanced rapid detection technologies. Expanded biomarker panels (e.g., host‐response proteins, metabolomic signatures, and exosomal miRNAs) have improved opportunities for multiplexed UTI diagnosis [62, 63]. Meanwhile, nanotechnology and chemically modified DNAzyme‐based strategies have enabled nanobiosensors with optical, magnetic, electrochemical, and mass‐based detection modes, allowing pathogen detection in urine within hours [64, 65]. These platforms showed promise for rapid and cost‐effective point‐of‐care diagnostics; however, challenges remain, including background leakage in multi‐component systems, limited large‐scale clinical validation, and practical barriers such as operator training and regulatory approval. Here, to circumvent these challenges, we developed a programmable unimolecular DNAzyme biosensor by integrating the allosteric detection module and catalytic module into one single‐stranded DNA molecule, which was denoted as SMART (sensitive and multiplex detection of ATP and miRNAs for UTI diagnosis).

Our previous work developed a similar unimolecular DNAzyme biosensor for serum miRNA detection [45]. However, it was restricted by a single miRNA biomarker for disease diagnosis, the requirement of miRNA extraction from biofluid, and limited diagnostic sensitivity and specificity. To solve these drawbacks, we further developed the unimolecular biosensor by expanding the species and quantity of biomarkers to fulfill simultaneous small molecule and multiple miRNA detection for UTI diagnosis without biomarker extraction with high sensitivity and specificity. The binding of targets to the detection module induces conformational changes for destabilizing the locking domains to enable processive invasion and cleavage of fluorescent reporters. Engineered as a self‐locked unimolecular entity, SMART eliminates stoichiometric precision requirements and split catalytic core designs that are inherent to multi‐component systems, which offers immediate advantages such as preserving DNAzyme's high cleavage efficiency and suppressing leakage‐induced background noise due to perfect stoichiometry and enhanced thermodynamic stability. The single‐strand self‐locking design of SMART simplifies stoichiometry, eliminating the complex concentration optimization required for multi‐component systems and reducing signal loss from incomplete assembly or conversion. In addition, SMART's single‐strand structure is entropically favored over intermolecular hybridization, offering superior thermodynamic stability that resists spontaneous opening and significantly reduces background leakage. SMART exhibited high sensitivity (∼pM for ATP; ∼fM for miRNAs) and multiplexing capability (∼4 markers) for the rapid (∼2.5 h) detection of UTI‐relevant markers in an extraction‐free, preamplification‐free, one‐pot, and isothermal manner. We demonstrated the clinical utility of the SMART platform through integrating it with an AI algorithm to establish a UTI diagnosis model via one‐pot detection of ATP and miRNAs directly from urine samples, which achieved exceptional diagnosis performance with 95.5% accuracy and 96.2% precision, showing great application potential that may be readily translated into clinics for the diagnosis of UTI and many other diseases.

## 2 Results and Discussion

### 2.1 Design Principle of SMART

**FIGURE 1:**
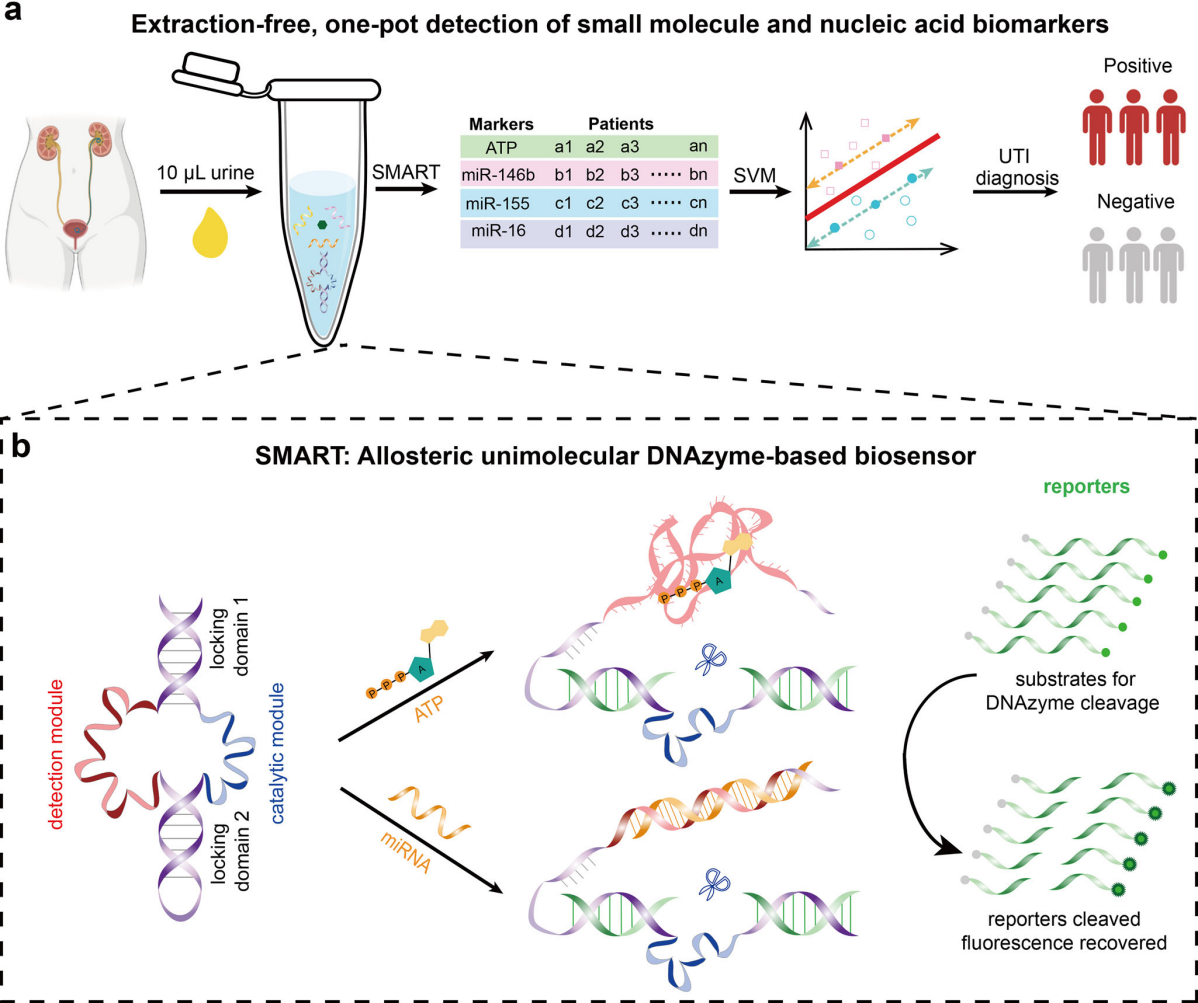
Sensitive and multiplex detection of small molecules and miRNA markers for UTI diagnosis based on SMART. (a) Overall workflow of SMART for UTI diagnosis. A 10 microliter urine sample was directly subject to SMART detection for four biomarkers (ATP, miR‐155, miR‐146, miR‐16) at ambient temperature within 2.5 h. The expression profiles were analyzed by the UTI diagnosis model to judge the infection status of the sample. (b) Working principle of SMART. The SMART system incorporates a target‐binding detection module and the 10–23 DNAzyme catalytic module into one single strand. The detection module is placed between two locking domains serving as a target‐recognition region via aptamer binding (for ATP) or sequence complementarity (for miRNAs). In the absence of targets, SMART remains inactive due to the two locking domains. Once ATP or miRNA binds to the detection module to induce conformational reconfiguration, SMART is activated to enable continuous cleavage of fluorescent reporters.

SMART is a self‐locked allosteric DNAzyme‐based biosensor incorporating multiple modules into a single DNA strand (Figure 1; Table S1). The detection module is a single‐stranded DNA of a specific sequence for target recognition and binding. The catalytic module is the 15‐nt‐long catalytic core of the DNAzyme for the cleavage of molecular reporters. 10–23 DNAzyme is used in SMART as it was reported to exhibit the highest cleaving efficiency of RNA substrates [28, 66, 67]. The two flanking arms of DNAzyme are sealed into two locking domains to inhibit its catalytic activity prior to target binding. Once the target binds to the detection module via aptamer recognition (for ATP) or sequence complementarity (for miRNA), the locking domains get partially opened to allow invasion of molecular reporters to initiate continuous cleavage and fluorescence accumulation. The length or strength of locking domains is essential to modulate the detection capability of SMART. A moderate locking strength is needed for SMART since high strength impedes activation, but weak strength may lead to signal leakage. This unimolecular feature of SMART eliminates the strict requirement for precise stoichiometric control of multiple DNA strands. Furthermore, unlike conventional split designs, SMART can preserve the efficient cleavage activity of the integrated catalytic core of DNAzyme for signal amplification and suppress background noise via energy‐favored conformational locking, both of which shall contribute to enhanced detection sensitivity. Finally, due to the high programmability of the detection module and the high specificity of reporter cleavage based on sequence complementarity, rational design of SMART systems can enable simultaneous multiplex detection of various markers in a one‐pot assay.

### 2.2 Design and Validation of SMART for ATP Detection

**FIGURE 2:**
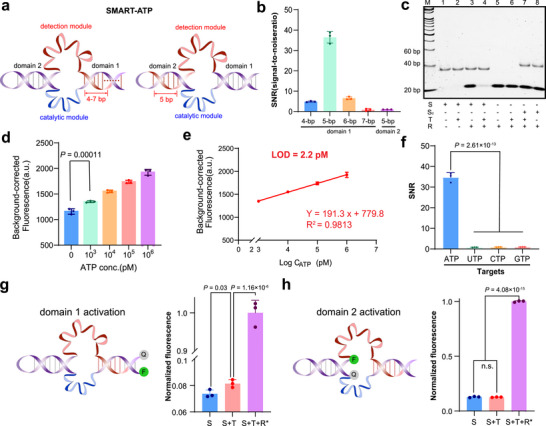
Design and validation of SMART for ATP detection. (a) Schematic illustration of SMART‐ATP. The detection module is composed of the ATP aptamer with 4–7 bp or 5 bp of its sequence inserted into domain 1 and domain 2, respectively. (b) SMART‐ATP of 5‐bp insertion in domain 1 exhibited the highest signal‐to‐noise ratio (SNR) for ATP detection. SNR was the ratio of fluorescence with ATP against that without ATP. (c) Native PAGE analysis of SMART‐ATP. Reporters were dramatically cleaved in the presence of ATP (Lane 4), verifying the detection capability of SMART. Lane M: 20 bp DNA ladder; Lane 1: SMART‐ATP; Lane 2: SMART‐ATP and ATP; Lane 3: SMART‐ATP and reporter; Lane 4: SMART‐ATP, ATP, and reporter; Lane 5: reporter; Lane 6: ATP and reporter; Lane 7: catalytic module mutational SMART‐ATP, ATP, and reporter; Lane 8: catalytic module mutational SMART‐ATP and reporter. S: SMART‐ATP; S_d_: disordered SMART‐ATP containing catalytic module mutant; T: ATP; R: reporter. (d) Background‐corrected fluorescence for detecting ATP of different concentrations. (e) Linear relationship of background‐corrected fluorescence for different ATP concentrations, demonstrating good quantification capability (R^2^ = 0.9813). The limit of detection (LOD) is calculated to be 2.2 pM. (f) SMART‐ATP exhibited high specificity toward ATP against structurally analogous targets (UTP, CTP, GTP). (g, h) FRET experiments to investigate the activation pathway of SMART‐ATP by examining the conformational change of domain 1 and domain 2 upon target and reporter binding. S: SMART‐ATP; T: ATP; R*: a DNA analog of the RNA reporter, whose ribonucleotide around the cleavage site was replaced by a deoxyribonucleotide. For detection experiments, SMART‐ATP: 10 nM; reporter: 500 nM; ATP: 100 µM or various concentrations for the LOD test. *P*‐values were calculated by a two‐tailed Student's *t*‐test. All experimental measurements are the mean ± SD with *n* = 3.

ATP is an important marker related to pathogen‐induced metabolism, which holds large potential for UTI diagnosis [6, 7]. The design of SMART for ATP detection (SMART‐ATP) was illustrated in Figure 2a, where the ATP aptamer [68, 69] was used to serve as the detection module. To pinpoint the optimal length of locking domains, we conducted a comprehensive computational study of various SMART‐ATP designs by using NUPACK [70]. Simulation results suggested that in order to maintain stably locked, the minimal length of domain 1 and domain 2 are 6‐bp and 4‐bp, respectively (Figure S2). To minimize signal leakage, we decided to set the length of locking domains as 7‐bp and 5‐bp, which we believe is thermodynamically stable but not too stable to inhibit SMART activation. For the initial design, the whole ATP aptamer sequence was incorporated into the detection module of SMART‐ATP, which, however, cannot be efficiently activated to enable reporter cleavage after adding ATP (Figure S3), which may be due to the fact that the binding of ATP to the aptamer could not induce a significant conformational change to destabilize the locking domains. Therefore, we next proposed to partially insert the ATP aptamer sequence (4 to 7‐bp) into locking domain 1 and 5‐bp into locking domain 2 to generate a stronger destabilization force upon ATP binding (Figure 2a; Figure S4). Experimental results confirmed the successful activation of SMART‐ATP, with the 5‐bp insertion in domain 1 design exhibiting the highest signal‐to‐noise ratio (SNR) for ATP detection (Figure 2b; Figure S5). For domain 1, the 4‐bp insertion may not be able to induce sufficient conformational change after ATP binding, while the 6‐bp and 7‐bp designs may impede the binding of ATP; both would fail to activate SMART. For domain 2, the 5‐bp insertion may not induce the desired conformational change after ATP binding. Therefore, the SMART‐ATP‐5‐bp design in domain 1 was chosen for subsequent ATP detection experiments. Native polyacrylamide gel electrophoresis (PAGE) was then used to further verify the performance of SMART‐ATP (Figure 2c). It was revealed that RNA reporters can only be cleaved in the presence of ATP (lane 4), proving the successful activation of SMART by ATP, consistent with the fluorescence assay. We next comprehensively examined various experimental parameters to pinpoint the optimal working conditions of SMART for ATP detection, including ATP incubation temperature, reporter cleavage temperature, ionic strength, etc. (Figure S7). It was found that SMART achieved the best detection performance when operated in the buffer containing 110 mm Mg^2+^ and 700 mm Na^+^ at room temperature.

Under the best operational conditions, the detection sensitivity and specificity of SMART‐ATP were assessed. For the determination of sensitivity, ATP with known concentrations ranging from 1 nm to 1 µm was subjected to SMART‐ATP detection (Figure 2d). There was a good linear correlation between fluorescence and logarithm of ATP concentrations (R^2^ = 0.9813) with the theoretical limit of detection (LOD) calculated to be 2.2 pm (Figure 2e) [6, 14, 15, 16, 71]. To examine the detection specificity of SMART‐ATP, cross‐reactivity was evaluated against structurally analogous small molecules (UTP, CTP, GTP) of ATP at equal concentrations (Figure 2f). As revealed, SMART‐ATP demonstrated high specificity for detecting ATP, exhibiting ∼35‐fold higher SNR compared to UTP, CTP, and GTP.

We next sought to investigate whether the operational mechanism of SMART‐ATP was as designed, where domain 1 is opened to serve as a toehold for RNA reporter invasion. Fluorescence resonance energy transfer (FRET) experiments were designed and conducted to probe the conformational states of SMART‐ATP before and after ATP binding. A pair of FRET molecules (FAM and BHQ1) was placed at the terminal of domain 1 (Figure 2g) or domain 2 (Figure 2h). Prior to activation, both domains remain locked, and FAM was quenched. Once the domain is opened, the fluorescence of FAM will be recovered due to the enlarged distance between FAM and the quencher, a good indicator for conformational reconfiguration at the molecular level. FRET experiments revealed that domain 1 was partially opened after ATP binding, as enhanced FAM fluorescence was observed. In contrast, FAM fluorescence of domain 2 had no enhancement after ATP binding, suggesting it remained fully sealed. The subsequent addition of the DNA mimic of RNA reporter led to further elevated fluorescence, suggesting that domain 1 was only partially opened by ATP. This study unambiguously confirmed the working mechanism of SMART‐ATP.

Furthermore, we investigated why the SMART‐ATP‐5‐bp design in domain 2 cannot be activated with the same design of the FRET experiments (Figure S6). Upon ATP addition, a remarkable FAM signal increase in the domain 2‐labeled probe, indicating substantial domain 2 opening, whereas only a slight increase was observed for domain 1. Importantly, subsequent addition of a DNA mimic of the RNA reporter did not lead to further fluorescence enhancement in either case, demonstrating that the ATP‐induced conformational change is insufficient to trigger large reporter invasion, leading to a low detection signal.

To validate the generalizability of our design, we replaced the ATP aptamer with the L‐lactate aptamer with the same SMART design and kept 5 bp inserted into domain 1 (Figure S8). Under the same reaction conditions, the performance of SMART‐L‐lactate was assessed. L‐lactate with concentrations ranging from 10 mm to 10 µm was subjected to detection. The results showed a good linear correlation between fluorescence and logarithm of L‐lactate concentrations (R^2^ = 0.9301) with the 3.6 µm limit of detection (LOD).

### 2.3 Design and Validation of SMART for miRNA Detection

**FIGURE 3:**
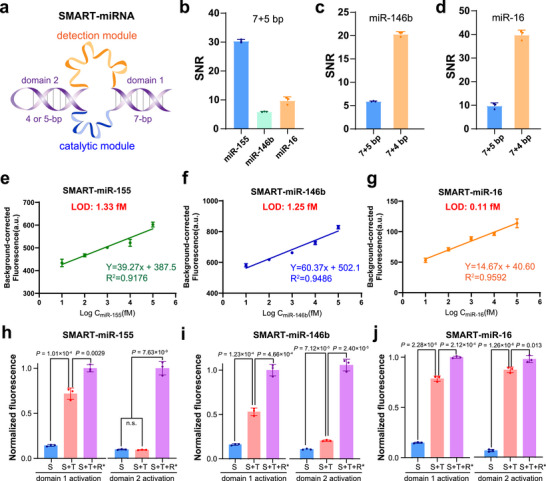
Design and validation of SMART for miRNA detection. (a) Schematic design of SMART‐miRNA. The detection module is a single‐stranded DNA that is fully complementary to the miRNA targets. (b) Detection of miR‐155, miR‐146b, miR‐16 by SMART‐miRNA designs with locking domains set as 7‐bp and 5‐bp. (c, d) SNR was significantly enhanced for miR‐146b and miR‐16 by shortening the length of domain 2 bp to 4 bp. (e–g) Determination of LOD for SMART‐miRNA against corresponding miRNA targets. (h–j) FRET experiments to investigate the activation pathway of SMART‐miRNA upon binding of miRNA targets. S: SMART‐miRNA; T: miRNA target; R*: a DNA analog of the RNA reporter. SMART‐miRNA: 10 nM; reporters: 500 nM; miRNAs: 10 nM or various concentrations for the LOD test. All experimental measurements are the mean ± SD with *n* = 3. *p* Values were calculated by a two‐tailed Student's *t*‐test.

After establishing the highly sensitive and specific SMART system for ATP detection, we extended SMART to miRNA (SMART‐miRNA) due to its large potential as a promising marker for UTI diagnosis [8, 9]. We selected miR‐155 and miR‐146b as diagnosis markers, which have been reported to exhibit elevated expressions in UTI patients [72, 73, 74]. miR‐16 is simultaneously detected as the endogenous housekeeping marker to truly reflect and quantify the expression levels of miR‐155 and miR‐146b. We initially adopted the SMART‐ATP design for SMART‐miRNA, where the length of domain 1 and domain 2 was set as 7 bp and 5 bp, respectively (Figure 3a). But the detection module was designed to be fully complementary to the miRNA target (Figure S9). We assume the formation of a rigid double helix after miRNA binding to the detection module would induce significant mechanical tension into the complex that would destabilize the locking domains to allow reporter invasion, which was confirmed by computational calculations to show that domain 1 was opened after miRNA binding to the detection module (Figure S10). Detection experiments revealed that SMART‐miRNAs can be successfully activated by their miRNA targets to produce significantly elevated fluorescent signals (Figure 3b), with SMART‐miR‐155 exhibiting great detection performance, whose SNR can be as high as ∼30. In comparison, SMART‐miR‐146b and SMART‐miR‐16 only achieved moderate SNR due to insufficient opening of locking domain 2 after miRNA binding to the detection module with low signal leakage (Figure S14). To further promote activation of SMART‐miR‐146b and SMART‐miR‐16, the length of domain 2 was shortened to 4‐bp to lower the energy barrier for reporter binding (Figures S11–S13). Although NUPACK predicted a higher hybridization efficiency for the 7+5 design, this calculation does not fully capture the complexity of the experimental system, and the final design was therefore determined based on experimental performance. The observed discrepancy may arise from differences between simulation and experimental conditions (e.g., pH, buffer composition and sequence features), as well as factors not fully considered in the model, including RNA transient conformations, target accessibility, and kinetic effects such as strand displacement efficiency, which may favor the 7+4 design in practice. As expected, both SMART‐miR‐146b and SMART‐miR‐16 showed significantly enhanced detection performance against their corresponding targets owing to substantial enhancement of reporter cleavage after miRNA invasion (Figure 3c,d). With the best SMART‐miRNA designs pinpointed, we then moved on to examine their detection sensitivity. miRNAs with gradient concentrations ranging from 10 fM to 10 pM were subject to SMART‐miRNA detection (Figures S16 and S17). There was good linear correlation between fluorescence and logarithm of miRNA concentrations with high coefficient of determination (0.9176 for miR‐155, 0.9486 for miR‐146b, 0.9592 for miR‐16), whose LOD was determined to be 1.33 fM for miR‐155 (Figure 3e), 1.25 fM for miR‐146b (Figure 3f), and 0.11 fM for miR‐16 (Figure 3g). SMART‐miRNA is among the highly sensitive methods for miRNA detection in a preamplification‐free manner, which is sufficient for detecting biological miRNAs whose concentration is typically in the fM‐pM range [51, 59]. To investigate the specificity of SMART‐miRNA, we introduced single‐base mutations at different positions along the miR‐146b sequence (position 1, 5, 9, 16, 19, and 21 from the 5' end) and tested its homologous family member miR‐146a (Figure S18). Both miR‐146a and all single‐nucleotide variants (SNVs) exhibited significantly lower fluorescence signals compared to the wild‐type (WT) miR‐146b, demonstrating that SMART can discriminate single‐nucleotide differences and homologous miRNA family members. We also observed position‐dependent discrimination, where SNVs with mutations closer to the 3' end showed better discrimination than those near the 5' end. This finding aligned with our proposed molecular unlocking mechanism, where 3'‐end mutations more impaired the target's ability to unlock domain 1, thereby preventing SMART activation more effectively.

We next sought to experimentally investigate whether the activation mechanism of SMART‐miRNA is as computationally simulated. Like SMART‐ATP, FRET experiments were utilized to study the conformational changes of two locking domains in SMART‐miRNAs (Figure S19). For SMART‐miR‐155, after miRNA binding, domain 1 was opened, with domain 2 remaining locked (Figure 3h). In comparison, both domains opened when miRNAs bound to the detection modules in SMART‐miR‐146b (Figure 3i) and SMART‐miR‐16 (Figure 3j), which was in good agreement with our rational designs of shortening domain 2 from 5‐bp to 4‐bp to lower its energy barrier for opening.

In comparison with conventional allosteric DNAzyme for ATP and miRNA detection based on split‐and‐resume and toehold‐mediated strategies (Table S2), SMART both exhibited a large improvement in detection performance. For signal‐to‐noise ratio, there was a 1.81‐ and 10.47‐fold increment for ATP detection (Figure S15a–c), and 2.53‐ and 1.50‐fold increment for miRNA detection (Figure S15d–f), respectively. Hence, the unimolecular SMART greatly solved the existing problems in the classical assays.

### 2.4 Multiplex Detection of ATP and miRNAs in One‐Pot by SMART

**FIGURE 4:**
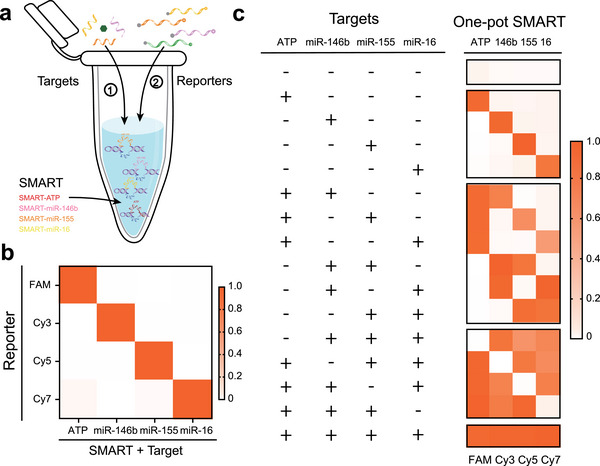
Multiplex detection of ATP and miRNAs in one‐pot by SMART. (a) Scheme for multiplex detection of ATP and miRNAs in one pot. After incubating ATP and miRNAs with SMART (step 1), corresponding reporters carrying different fluorophores were added for cleavage (step 2). (b) Validation of reporter cleavage specificity for 4 different SMART systems. Fluorophores: FAM for ATP, Cy3 for miR‐146b, Cy5 for miR‐155, Cy7 for miR‐16. Scale bar: normalized intensity. (c) Validation of orthogonality of SMART systems for detecting 4 different targets in one‐pot. Scale bar: normalized intensity. SMART: 10 nM each, reporters: 500 nM each, ATP: 10 µM, miRNAs: 10 nM each. All experimental measurements are the mean ± SD with *n* = 3.

Since our goal was to realize one‐pot detection of ATP and miRNA targets, we then examined and validated the multiplex detection specificity and orthogonality of SMART systems prior to detecting real‐world clinical samples (Figure 4a). We first investigated the reporter cleavage specificity of SMART, which was independently activated by its targets, and then incubated with the mixture of four fluorescent RNA reporters. All SMART systems exhibited high cleavage specificity against their corresponding RNA reporters with no apparent crosstalk observed (Figure 4b). Furthermore, we tested the detection orthogonality of SMART with all targets present in one pot. Co‐incubation of four distinct reporters in the presence of single or multiple targets demonstrated highly specific activation and cleavage of four SMART systems (Figure 4c). These results confirmed that SMART was capable of detecting multiplex molecular targets in one pot with high specificity dependent on both the specificity of reporter cleavage and the detection loop.

### 2.5 Direct Detection of ATP and miRNAs in Urine for the Rapid Diagnosis of UTI

Before applying SMART in urine for UTI diagnosis, we tested the quantitative accuracy of ATP and miRNAs in 10% urine matrices, whose proportion was the same as expected clinical sample detection, across three concentration levels (high‐concentration group with 100 µm ATP, 10 nm miRNAs each; mid‐concentration group with 1 µm ATP, 100 pm miRNAs each; low‐concentration group with 10 nm ATP, 1 pm miRNAs each). The recovery efficacy ranged from 87% to 110% across all targets and concentration levels, falling well within the acceptable range (Figure S21). Hence, SMART maintains reliable quantitative accuracy in complex urine matrices.

**FIGURE 5:**
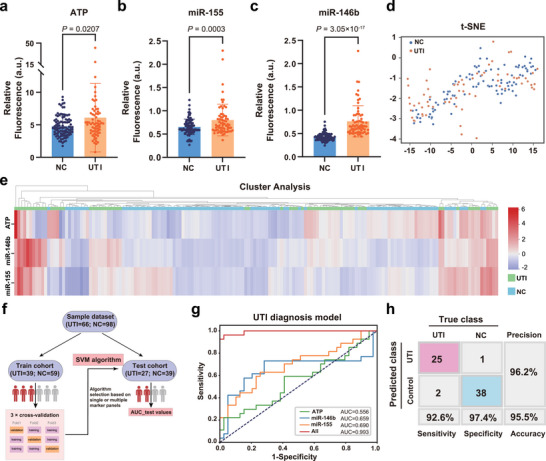
Direct detection of ATP and miRNAs in urine for the rapid diagnosis of UTI. (a) Bar plots of the expression levels of ATP for UTI patients (UTI, *n* = 66) and non‐infected controls (NC, *n* = 98). (b, c) Relative expression of miR‐155 and miR‐146b against the housekeeping gene miR‐16 for UTI patients and controls. (d) Unsupervised t‐SNE dimensionality reduction analysis of clinical samples. Each point represented one sample. (e) Unsupervised clustering heatmap of clinical samples based on marker expression levels. Scale bar: z‐score normalization across sample groups. (f) Scheme for machine learning to establish the UTI diagnosis model. (g) ROC curves and AUC analysis of the performance of diagnosis models based on single or all markers. (h) Confusion matrix for UTI diagnosis by using all markers. All experimental measurements are the mean ± SD with *n* = 3. *p*‐values were calculated by a two‐tailed Student's *t*‐test.

To validate the clinical utility of SMART for UTI diagnosis, a total of 164 urine samples, including 66 UTI patients and 98 non‐infected controls (NC) were collected and subject to SMART detection of ATP and miRNA markers (Table S3). Owing to its high sensitivity, urine samples were directly subject to SMART detection after a heating step for denaturing potential interfering nucleases while maintaining biomarker integrity (Figure S20) without marker extraction or preamplification of miRNAs. The measurement of ATP and three miRNA targets was conducted in one‐pot with a total of 2.5 h of operation, whose relative abundance was then profiled (Figure 5a–c). To be noted, miR‐16 was used as the housekeeping gene for normalizing the expression levels of other miRNA targets, which is a well‐accepted practice in the field to truly reflect the expression changes of miRNAs [75, 76, 77]. Despite high interpatient heterogeneity, UTI patients exhibited significantly elevated expressions for all markers compared to the NC groups. However, both T‐distributed stochastic neighbor embedding (t‐SNE) dimensionality reduction analysis (Figure 5d) or unsupervised clustering (Figure 5e) failed to clearly segregate UTI and NC individuals. The large overlap of UTI and NC may be because of different UTI severity, large variation of biomarker expression among NC, self‐administered antibiotics for some patients, and unpreserved global distance information for t‐SNE. The unsegregated results prompted the need for a more advanced machine learning‐based classifier. Employing a support vector machine with linear kernel (SVM‐Linear), we partitioned clinical samples into training (60%, n = 98; 39 UTI and 59 NC) and testing (40%, *n* = 66; 27 UTI and 39 NC) cohorts for establishing the diagnosis model (Figure 5f). The diagnostic performance of the model based on single or all markers was evaluated by plotting the receiver operating characteristic (ROC) curves and analyzing the area under the curve (AUC) value. Although single markers achieved moderate performance in UTI diagnosis (ATP: 0.556; miR‐146b: 0.659; miR‐155: 0.690), the combination of three markers significantly improved the diagnosis performance, with an AUC value of 0.993 (Figure 5g). Specifically, SMART exhibited a diagnosis sensitivity of 92.6%, specificity of 97.4%, along with an overall accuracy of 95.5% (Figure 5h). To assess the robustness of the model, we performed 1000 independent random train‐test splits with an AUC value of 0.991 ± 0.009, sensitivity of 0.9420 ± 0.034, and overall accuracy of 0.944±0.029 (Table S4). In general, SMART can surpass clinical culture‐based methods in speed, sensitivity, and reliability for UTI diagnosis. We anticipate that expanding the marker panel of SMART and validating its performance in larger multi‐center cohorts may further enhance its UTI diagnosis performance and promote its clinical translation.

## 3 Conclusion

In this work, we developed SMART, a DNAzyme‐based biosensor for enabling highly sensitive and specific detection of multiplex markers in one pot. SMART is designed as a unimolecular self‐locked system consisting of an allosteric detection module and a catalytic module. The binding of molecular targets to the detection module induced a conformational change of the complex to activate the catalytic activity of the DNAzyme for cleaving RNA substrates. SMART exhibited great sensitivity and specificity in detecting ATP and miRNAs simultaneously, which demonstrated great potential in clinical application for diagnosing UTI with high accuracy. SMART has several distinct characteristics compared to existing molecular detection methods. First, SMART is highly sensitive, exhibiting pM sensitivity for ATP and fM sensitivity for miRNAs. With such a high sensitivity, SMART can directly detect markers in urine in an extraction‐free, preamplification‐free, and rapid (∼2.5 h) manner. Secondly, owing to the sequence specificity of DNAzyme for substrate cleavage, SMART holds high specificity and multiplexing capability for detecting various targets simultaneously. Thirdly, SMART doesn't require expensive enzymes, rendering it highly cost‐effective (Table S5). Per calculation, the cost of SMART for one marker can be as low as $0.02, thus only $0.08 for a single UTI test. Taken together, we believe SMART can serve as a highly potential technical platform for detecting various biomarkers that may be translated toward clinical UTI diagnosis and beyond after expanding the marker panel of SMART and validating its performance in larger multi‐center cohorts.

Owing to the overlap of fluorescence spectra, the maximum throughput of detecting biomarkers based on a simple fluorescence readout is four. To further increase detection throughput based on allosteric DNAzyme, there are two main strategies to be developed. First, systematically designing reporters and fluorophore position achieves fluorescence microscopy readout. Second, regulating DNAzyme cleavage activity and combining multiple types of DNAzyme, such as 10–23, 8–17E, and 13PD1, fulfills higher detection throughput by sequencing readout.

### 3.1 Ethical Statement

Prior to study, written informed consent was obtained from all participants. All urine samples were collected from Renji Hospital, Shanghai Jiao Tong University School of Medicine (Shanghai, China). The study was approved by the Ethics Committee at Renji Hospital, School of Medicine, Shanghai Jiao Tong University (Approval No. KY2025‐010‐A). All procedures were conducted in accordance with these approved guidelines.

## Conflicts of Interest

The authors declare no conflict of interest.

## Supporting information




**Supporting File**: advs75417‐sup‐0001‐SuppMat.docx.

## Data Availability

The data that support the findings of this study are available in the supplementary material of this article.
