# Supplementary material for: Allosteric DNAzyme‐Enabled Sensitive and Multiplex Detection of Biomarkers for Rapid Diagnosis of Urinary Tract Infections
Source: Adv Sci (Weinh). 2026 Apr 20;13(40):e75417. doi: 10.1002/advs.75417 (PMC13335575; doi:10.1002/advs.75417)
Supplement: Supplementary file 1 — Supporting File: advs75417‐sup‐0001‐SuppMat.docx. [file ADVS-13-e75417-s001.docx]

**Supporting Information**

Allosteric DNAzyme-Enabled Sensitive and Multiplex Detection of Biomarkers for Rapid Diagnosis of Urinary Tract Infections

Yanzhe Shen^1#^, Chenzhi Shi^1#^, Xiaowei Ma^1#^, Pengfei Hou^1^, Haomin Zhang^1^, Juanxiu Qin^1^, Li Pan^1^, Guilin Li^2^, Lifei Gao^2^, Qian Ma^3^, Donglei Yang^1^*, Min Li^1^*, Pengfei Wang*

^1^ Institute of Molecular Medicine, Department of Laboratory Medicine, Shanghai Key Laboratory for Nucleic Acid Chemistry and Nanomedicine, Renji Hospital, School of Medicine, Shanghai Jiao Tong University, Shanghai 200127, China.

^2^ Autobio Diagnostics Co., Ltd., NO.199 15th Ave, National Eco & Tech Zone, Zhengzhou, Henan 450016, China.

^3^ Intellinosis Biotechnology Co., Ltd., Shanghai, 201112, China.

# Equal contribution

Correspondence: pengfei.wang@sjtu.edu.cn; rjlimin@shsmu.edu.cn; dongleiyang@shsmu.edu.cn

**Materials and Methods.**

**Overview of Assays.**

Sequences of all oligonucleotides are provided in Table S1. All oligonucleotides were purchased from Bioligo or Sangon Biotech and purified by high-performance liquid chromatography (HPLC). Each strand was dissolved in DEPC-treated water (Sangon, B501005) and quantified using a Thermo Scientific NanoDrop spectrophotometer to achieve a final stock concentration of 20 μM. All TE buffers used in experiments were prepared by diluting a 20×TE stock solution (Thermo Fisher Scientific, T11493), which contained 200 mM Tris-HCl (pH 7.5), 20 mM EDTA, and was certified RNase-free. Additionally, all oligonucleotides modified with fluorescent reporter groups were handled under light-protected conditions during preparation and storage.

The SMART assay was performed as follows: Initially, allosteric DNAzymes (SMART) were diluted in the optimized assay buffer (4 × TE, 110 mM Mg^2+^, 700 mM Na^+^). Then the diluted SMART was denatured at 95 °C for 3 minutes, followed by gradient annealing from 95 °C to 4 °C over 91 cycles (20 s per cycle, 1 °C decrement after a cycle) The annealed products were incubated with the target analyte at 25 °C for 30 minutes. Subsequently, 10 μL of an excess concentration of the fluorescent reporter strand was added (usually 500 nM in final concentration), and fluorescence measurements were initiated at 25 °C. To precisely monitor the reaction dynamics, fluorescence intensity was recorded every 70 seconds for a total duration of 2 hours. Fluorescence kinetic measurements were conducted using a Biotek Synergy H1 multimode microplate reader, with the entire reaction system assembled in WHB-96-02 black 96-well plates under a standardized 100-μL reaction volume. For signal correction, a background subtraction method was employed. Fluorescence intensity values from experimental wells were corrected by subtracting values measured in blank controls containing only buffer and reporter strand, yielding the net fluorescence signal arising specifically from target binding.

**NUPACK**^[1,2]^ **simulation**

Secondary structures and corresponding Gibbs free energies for both SMART and its target complex were calculated using NUPACK software at 25 °C; 0.7 M Na^+^; 0.11 M Mg^2+^ as model options, using DNA as the nucleic acid type. For SMART calculations, strand number and maximum complex size were set to 1. For the SMART-target complex, these parameters were set to 2 strands and size 3, respectively. Ratio of SMART:target equals to 1:1. Ratio of SMART:target:reporter equals to 1:1:50.

**Optimizing experimental conditions for SMART**

To achieve rapid, sensitive, and simultaneous detection of ATP and miRNAs, we optimized the reaction conditions using an excess of ATP target (100 μM final concentration) with SMART DNAzymes maintained at 10 nM. Given the critical influence of incubation temperature on both the thermodynamic stability of SMART and target-binding complexes, optimization experiments were performed across a range of temperature (4 °C, 16 °C, 25 °C, 37 °C) and incubation time (0, 0.5 h, 1 h, 1.5 h, 2 h, 2.5 h, 3 hours). Similarly, as the cleavage activity of SMART DNAzymes is highly dependent on divalent cation concentration, Mg^2+^ concentration in the buffer was systematically varied, incrementally increased from 30 mM to 170 mM. Fluorescence was measured over 2 hours at room temperature for reactions containing 10 nM SMART and 500 nM reporter substrate, both with (F) and without (F_0_) target. Experimentally, we observed that monovalent cation concentration also significantly impacted SMART-target complex stability. Consequently, Na^+^ concentration was similarly optimized, ranging from 100 mM to 1300 mM final concentration, following the same experimental protocol. Upon establishing the optimized conditions, the ATP target was replaced with miRNAs (miR-155, miR-146b, miR-16) to validate the applicability of the optimized protocol for simultaneous one-pot detection of multiple targets. A detection duration of 2 hours was selected, with fluorescence measurements taken every 70 seconds. All measurements were conducted in a multimode microplate reader, employing target-specific excitation and emission wavelengths (ATP: excitation wavelength: 485 nm, emission wavelength: 528 nm, Gain 100; miR-146b: excitation wavelength: 540 nm, emission wavelength: 580 nm, Gain 100; miR-155: excitation wavelength: 636 nm, emission wavelength: 667 nm, Gain 100; miR-16: excitation wavelength: 740 nm, emission wavelength: 785 nm, Gain 100).

**Native polyacrylamide gel electrophoresis (PAGE)**

Nucleic acid gel electrophoresis allows for the separation and discrimination of nucleic acid fragments based on molecular length and charge. Under an applied electric field, negatively charged nucleic acids migrate through a polyacrylamide gel matrix at rates inversely proportional to their size, enabling separation. 10% PAGE gels were prepared by combining 1875 μL of 40% acrylamide solution, 1500 μL of 5 × TBE buffer, 50 μL of 10% ammonium persulfate (APS), and 6 μL TEMED, with DEPC-treated water added to a final volume of 7 mL; gels were allowed to polymerize at room temperature.

Samples for electrophoresis were prepared identically to those for fluorescence measurements: 200 nM annealed SMART (DNAzymes), 100 μM target ATP, and 1 μM reporter strand were co-incubated in optimized assay buffer (4 × TE, 110 mM Mg^2+^, 700 mM Na^+^) at 25°C for 2.5 hours. Following ionic strength normalization across samples, loading buffer (1 × TBE) was added proportionally. Here was the PAGE loading scheme (10 μL per lane): Lane M: 20 bp DNA ladder; Lane 1: SMART-ATP; Lane 2: SMART-ATP and ATP; Lane 3: SMART-ATP and reporter; Lane 4: SMART-ATP, ATP, and reporter; Lane 5: reporter. S: SMART-ATP; T: ATP; R: reporter. Electrophoresis was performed at 120 V for 50 minutes at room temperature. Gels were subsequently stained with GelRed (41003, Biotium) for approximately 5 minutes and imaged using an Amersham Imager 680 RGB (GE Healthcare) to analyze band intensity and positional shifts.

**Screening and examining detection capability of SMART by using synthetic miRNAs**

For screening SMART designs, 100 nM SMART was first annealed in 1 × SMART buffer (4 × TE; 110 mM Mg^2+^; 700 mM Na^+^). Subsequently, 10 μL of the annealed SMART (100 nM in 1× SMART buffer), 10 μL miR-146b (100 nM in 1 × SMART buffer), and 10 μL of excess reporter strand (5 μM in 1 × SMART buffer) were combined with 70 μL of 1 × SMART buffer to achieve a final reaction volume of 100 μL. Final concentration of SMART, miRNA, and reporter were 10 nM, 10 nM, and 500 nM, respectively.

Following a 2 hour incubation at room temperature, fluorescence was measured using a multimode microplate reader, employing miRNA-specific parameters (miR-146b: excitation wavelength: 540 nm, emission wavelength: 580 nm, Gain 100; miR-155: excitation wavelength: 636 nm, emission wavelength: 667 nm, Gain 100; miR-16: excitation wavelength: 740 nm,emission wavelength: 785 nm, Gain 100).

To assess detection sensitivity, SMART was firstly annealed in 1 × SMART buffer. Then 10 μL of the annealed SMART (100 nM in 1× SMART buffer) was mixed with 10 μL of target analyte at varying concentrations (10× final concentration in 1× SMART buffer) and 10 μL of excess reporter strand (5 μM in 1× SMART buffer) in 70 μL of 1× SMART buffer. After a 2-hour incubation at room temperature, fluorescence was measured using the same multimode microplate reader and the miRNA-specific wavelength and gain settings as above.

**Comparison assay with traditional allosteric DNAzymes**

Toehold-mediated allosteric DNAzyme:

For ATP detection, all oligonucleotide sequences and assay workflows were according to being published by Zhang, Q.L. et al.^[3]^. 100 nM ATP-Output-DNAzyme and 100 nM ATP-Complementary-DNAzyme were annealed in Tris-HCl buffer with 20 mM Tris-HCl (pH 7.5), 0.1 mM EDTA, 100 mM NaCl and 20 mM MgCl_2_. The mixture was heated to 95 °C for 5 min and gradually cooled to 20 °C at a rate of 1 °C min^-1^. 90 μL of the operating solutions were prepared to contain 20 nM DNAzyme-ATP-toehold strand, 10 nM duplex consisting of ATP-Output-DNAzyme and ATP-Complementary-DNAzyme, and 500 nM substrate-ATP-toehold strand incubated in buffer with 20 mM Tris-HCl (pH 7.5), 0.1 mM EDTA, 100 mM NaCl and 20 mM MgCl_2_ at room temperature for 30 min. Finally, 10 μL of 100 μM ATP target was added and Fluorescence was measured at 528 nm in real time at room temperature for 2 h with excitation at 485 nm. Baseline controls were run out of ATP.

For miRNA detection, all oligonucleotide sequences and assay workflows were according to being published by Peng, H. et al.^[4]^ 100 nM DNAzyme-toehold-miR-155 strand and 100 nM locking-strand (lock6-miR-155) were annealed in Tris-acetate buffer with 25 mM Tris-acetate buffer (pH 8.0) and 200 mM NaCl. The mixture was heated to 75 °C and gradually cooled to 4 °C at a rate of 1.2 °C min^-1^. 95 μL of the operating solutions were prepared to contain 10 μL of 10 nM target sequence (miR-155, or no target sequence in parallel experiments to serve as reagent blanks), 10 μL of annealed mixture (final concentration: 10 nM DNAzyme-toehold-miR-155 strand and locking-strand) and 500 nM substrate-miR-155-toehold-/FAM/ in 25 mM Tris-acetate buffer (pH 8.0) and 200 mM NaCl. After incubation at room temperature for 20 min, a Mn^2+^ solution (5 μL, 10 mM) was added to initiate the operation of the motor. Fluorescence was measured at 528 nm in real time at room temperature for 2 h with excitation at 485 nm.

Split-and-resume allosteric DNAzyme:

For miRNA detection, all oligonucleotide sequences and assay workflows were according to being published by [Yuqiang Hu](https://onlinelibrary.wiley.com/authored-by/Hu/Yuqiang) et al.^[5]^ 100 nM split-DNAzyme-miR-155 and 100 nM effector strands (miR-155-5p) were first annealed in Tris-HCl buffer with 25 mM Tris-HCl (pH 7.5) and 200 mM NaCl. The mixture was heated to 85 °C and gradually cooled to 25 °C at a rate of 1 °C min^-1^. 10 μL of annealed mixture (final concentration: 10 nM split-DNAzyme-miR-155 and effector strand) were added in buffer with 25 mM Tris-HCl (pH 7.5) and 200 mM NaCl. 500 nM FAM-split-reporter strand with 50 mM Mg^2+^ cofactor were then introduced to initiate RNA cleavage, bringing the total volume to 100 µL. Fluorescence was measured at 528 nm in real time at 37 °C for 2 h with excitation at 485 nm.

For ATP detection, all oligonucleotide sequences and assay workflows were according to being published by [Yuqiang Hu](https://onlinelibrary.wiley.com/authored-by/Hu/Yuqiang) et al.^[5]^. 10 nM split-DNAzyme-ATP and 100 μM ATP were mixed in Tris-HCl buffer with 25 mM Tris-HCl (pH 7.5) and 200 mM NaCl. 500 nM FAM-split-reporter strand with 50 mM Mg^2+^ were then introduced to initiate RNA cleavage, bringing the total volume to 100 µL. Fluorescence was measured at 528 nm in real time at 37 °C for 2 h with excitation at 485 nm.

**Specific cross-reactivity assessment among SMART designs**

To assess specificity of the four SMART designs in one-pot (ATP, miR-155, miR-146b, and miR-16), a 4×4 cross-reactivity matrix was constructed by systematically challenging each fluorescent reporter with all four targets. Initially, 100 nM SMART specific to four targets were individually prepared. Defined reaction wells contained: 10 μL of a single annealed SMART (10 nM final concentration), 10 μL of its cognate or non-cognate target (10 nM miRNAs or 10 μM ATP) and 10 μL of corresponding excess reporter strand (500 nM final concentration) in 70 μL 1× SMART buffer (100 μL total volume). All reactions were assembled in WHB-96-02 black 96-well plates under standardized conditions.

To validate orthogonality of SMART for detecting multiple small molecule and miRNA targets in a one-pot assay. Individually annealed 1 μM SMART corresponding to ATP, miR-146b, miR-155, and miR-16 in 1 × SMART buffer (4 × TE, 110 mM Mg^2+^, 700 mM Na^+^) were then combined to create a master mix containing 100 nM of each 4 SMART (SMART-ATP, SMART-miR-146b, SMART-miR-155, and SMART-miR-16). Per well in WHB-96-02 plates, the mixture was challenged with: 20 μL reporter strand cocktail (5 μM final per dye-conjugated reporter: FAM-ATP, Cy3-miR-146b, Cy5-miR-155, Cy7-miR-16) and 10 μL combinatorial target sets (single/double/triple/quadruplet combinations) in 60 μL 1× SMART buffer (100 μL total). Following 2 hours incubation at room temperature, wavelength-resolved fluorescence measurements (Biotek Synergy H1) quantified cross-reactivity.

**Urine ATP and miRNAs detection by SMART for UTI diagnosis**

Samples were registered with subject information and assigned unique identifiers. For each sample, 20 μL of urine was collected into an eppendorf microcentrifuge tube and centrifuged at 12,000 rpm (4 °C) for 10 minutes. Before analysis, samples were heated at 70 °C for 5 minutes to denature potential interfering nucleases. All reaction mixtures were supplemented with DNase and RNase inhibitors to prevent nucleic acid degradation.

First, 100 nM SMART corresponding to ATP, miR-146b, miR-155, and miR-16 were individually annealed in optimization buffer (4 × TE, 110 mM Mg^2+^, 700 mM Na^+^). Subsequently, 10 μL of each annealed target-specific SMART (10 nM final concentration: SMART-ATP, SMART-miR-146b, SMART-miR-155, and SMART-miR-16 in 1 × SMART buffer), 10 μL of urine sample, and 20 μL of corresponding excess reporter strand (500 nM final concentration per reporter in 1× SMART buffer) were combined with 60 μL of 1× SMART buffer to achieve a final reaction volume of 100 μL. Following a 2-hour incubation at room temperature, fluorescence was measured using a multimode microplate reader, employing ATP and miRNA-specific parameters. (ATP: excitation wavelength: 485 nm, emission wavelength: 528 nm, Gain 100; miR-146b: excitation wavelength: 540 nm, emission wavelength: 580 nm, Gain 100; miR-155: excitation wavelength: 636 nm, emission wavelength: 667 nm, Gain 100; miR-16: excitation wavelength: 740 nm, emission wavelength: 785 nm, Gain 100). LOD was calculated according to IUPAC guidelines as: LOD = mean of blanks + 3× standard deviation of blanks, with blanks defined as the reporter and SMART only fluorescence in buffer (without target analyte).

**Machine learning model for UTI diagnosis**

In this study, machine learning model was trained using the scikit-learn library (version v1.6.1) in Python. The following steps outline the model training and evaluation process: (1) Feature Normalization: All features were standardized using StandardScaler from scikit-learn to ensure that all input variables had zero mean and unit variance, which is critical for optimal SVM performance. (2) The dataset was split into training and testing subsets using train_test_split from scikit-learn, with 60% of the data used for training and 40% for testing. The random state was fixed to ensure reproducibility of results. (3) Hyperparameters were rigorously optimized on the training set using grid search with stratified 3-fold cross-validation. The classification threshold was selected by maximizing the ROC curve on the training data. This process was repeated independently for each iteration to ensure generalizability and prevent overfitting. (4) The performance of the trained model was evaluated on the test set using various metrics, including accuracy, precision, sensitivity, F1-score and AUC **(Supplementary Table S4)**. The classification_report and confusion_matrix functions from scikit-learn were used to provide a detailed assessment of the model's classification performance.

**Statistical analysis**

Statistical analyses were performed using GraphPad Prism. The fluorescence signal-to-noise ratio (SNR) of SMART was calculated from background-subtracted (F_0_) measurements (S - F_0_) / (N - F_0_), where S and N represent signals with and without target at 2 hours, respectively. Background-corrected fluorescence signals were subjected to linear regression against logarithmic target concentrations to establish correlation models. Inter-group comparisons between patient and control cohorts employed two-tailed unpaired t-tests. Diagnostic performance was evaluated through receiver operating characteristic (ROC) curve analysis, with area under the curve (AUC) values quantified at 95% confidence intervals.

**Ethical Statement**

Prior to study, written informed consent was obtained from all participants. All urine samples were collected from Renji Hospital, Shanghai Jiao Tong University School of Medicine (Shanghai, China). The study was approved by the Ethics Committee at Renji HospitaI, SchooI of Medicine, Shanghai Jiao Tong University (Approval No. KY2025-010-A). All procedures were conducted in accordance with these approved guidelines.

**
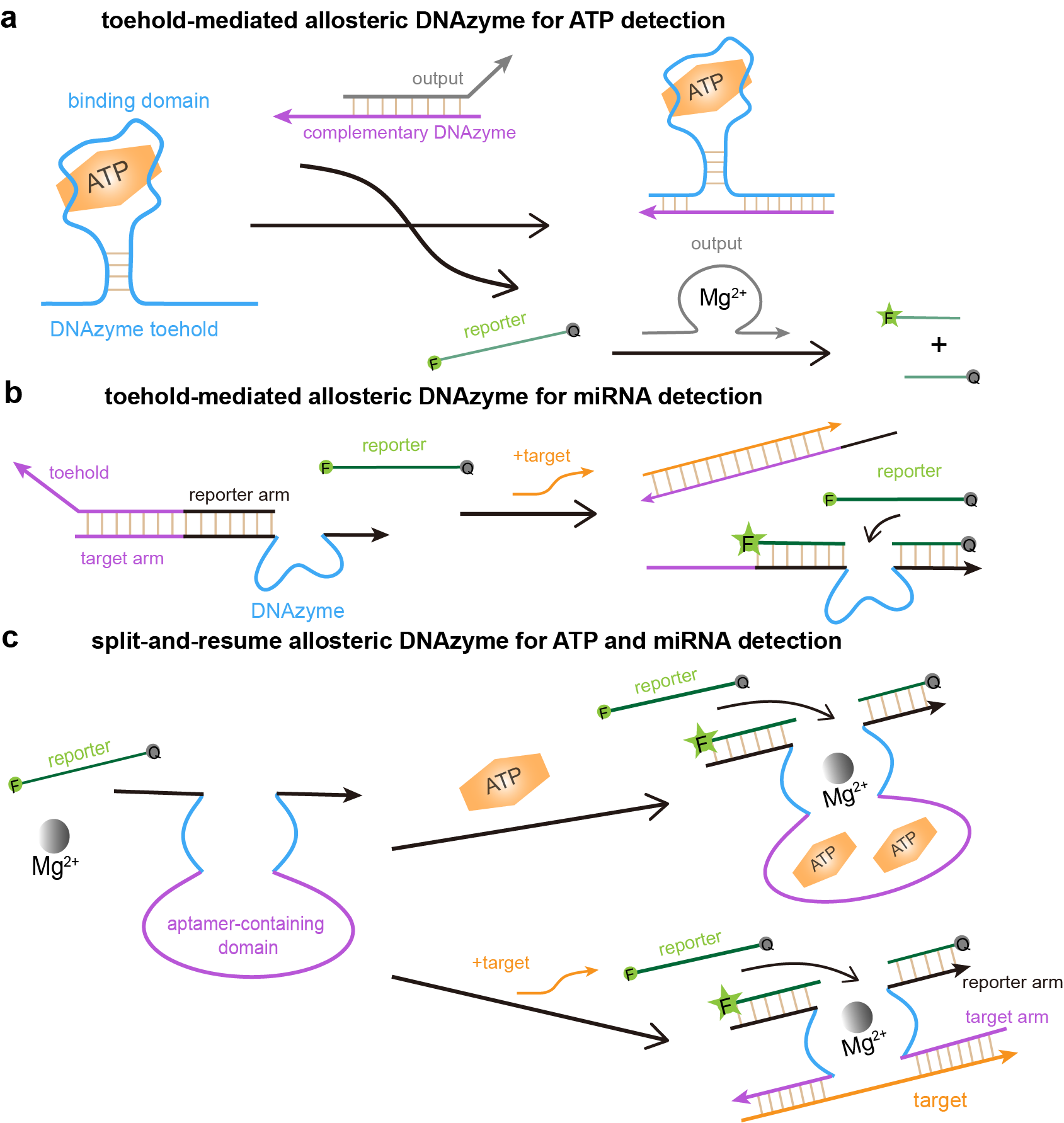
**

**Figure S1. Conventional allosteric DNAzyme biosensors with multicomponent molecular complexes**. **a-b,** Toehold-mediated strand displacement enables the allosteric regulation of DNAzyme function^[3,6]^. **c**, split-and-resume allosteric DNAzyme by splitting and resuming the catalytic core upon target binding^[5]^.

**

**

**Figure S2. Secondary structures of SMART-ATP designs with various locking domain length predicted by NUPACK. Domain 1: 8 bp to 5 bp; domain 2: 8 bp to 3 bp. SMART designs with nonlocked domains (domain 1 or domain 2 or both) are highlight by dashed red box.**

**
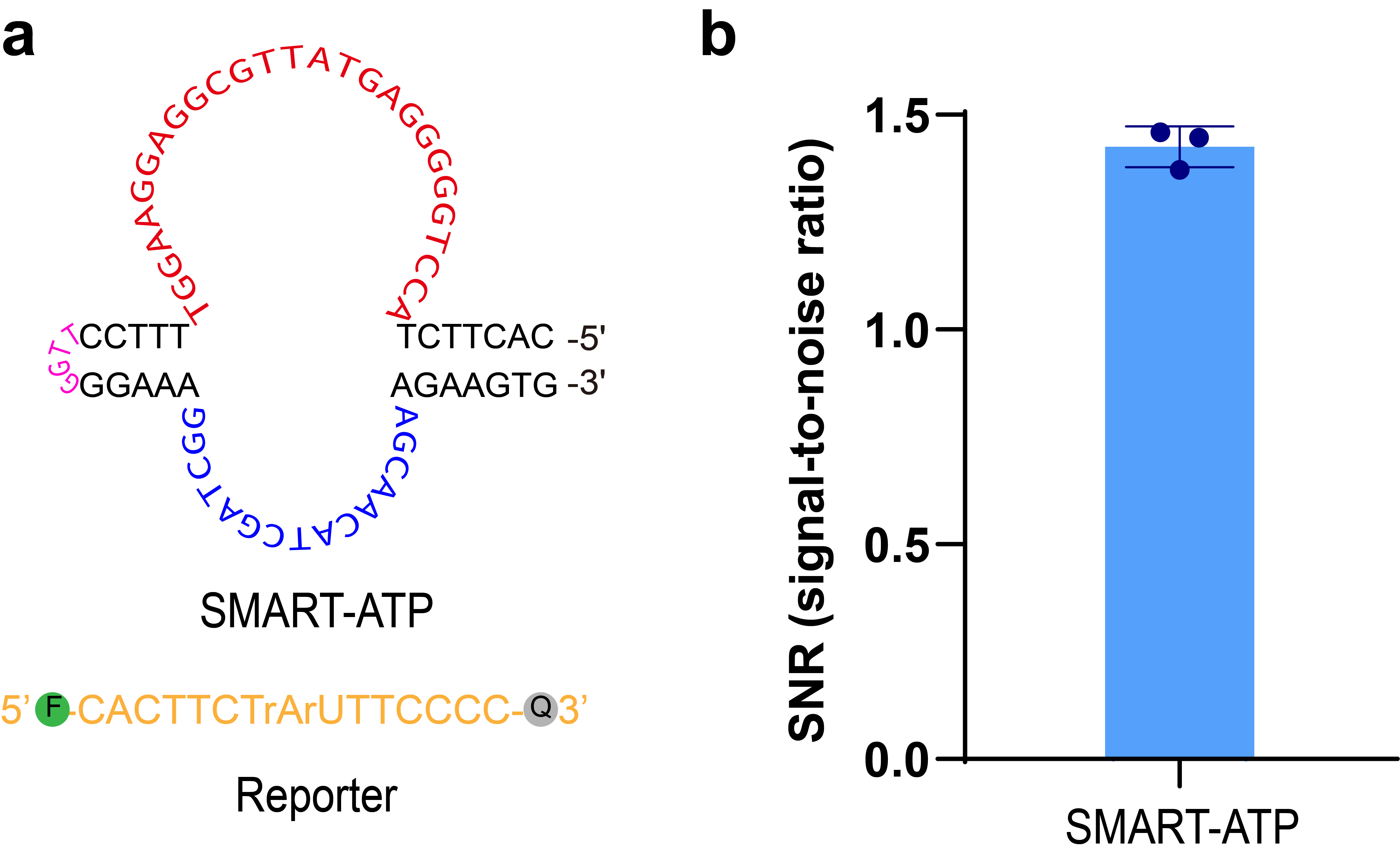
**

**Figure S3. Design and validation of SMART-ATP constructed from 10-23 DNAzyme and ATP aptamer. a,** SMART-ATP design with full-length ATP aptamer as the detection module. Domain 1 is 7-bp, domain 2 is 5-bp. **b,** Fluorescence of 10 nM annealed SMART-ATP after incubating with 100 μM ATP molecules. Signal-to-noise ratio (SNR) was the ratio of fluorescence with ATP against without ATP.

**

**

**Figure S4. Revised SMART-ATP designs with aptamer sequence (4 to 7-bp) inserted into locking domain 1 and (5-bp) inserted into locking domain 2.**

**

**

**Figure S5. Screening SMART-ATP designs. a-e**, Systematic length-optimization of ATP aptamer fusion lengths (4 to 7-bp) to domain 1 and (5-bp) to domain 2 for ATP detection. The reaction mixture contained 10 nM SMART-ATP and 100 μM target ATP. Time-dependent cleavage of diverse sequence complementary substrates over 2 hours. All experimental measurements are mean ± standard deviation (SD) with n = 3.

**

**

**Figure S6. SMART-ATP for aptamer sequence 5-bp inserted into locking domain 2. a,b,** FRET experiments to investigate the activation pathway of SMART-ATP by examining the conformational change of domain 1 and domain 2 upon target and reporter binding. S: SMART-ATP; T: ATP; R*: a DNA analog of the RNA reporter, whose ribonucleotide around cleavage site was replaced by deoxyribonucleotide. For detection experiments, SMART-ATP: 10 nM; reporter: 500 nM; ATP: 100 μM or various concentrations for the LOD test. P values were calculated by two-tailed Student’s T test. All experimental measurements are the mean ± SD with n = 3.

**
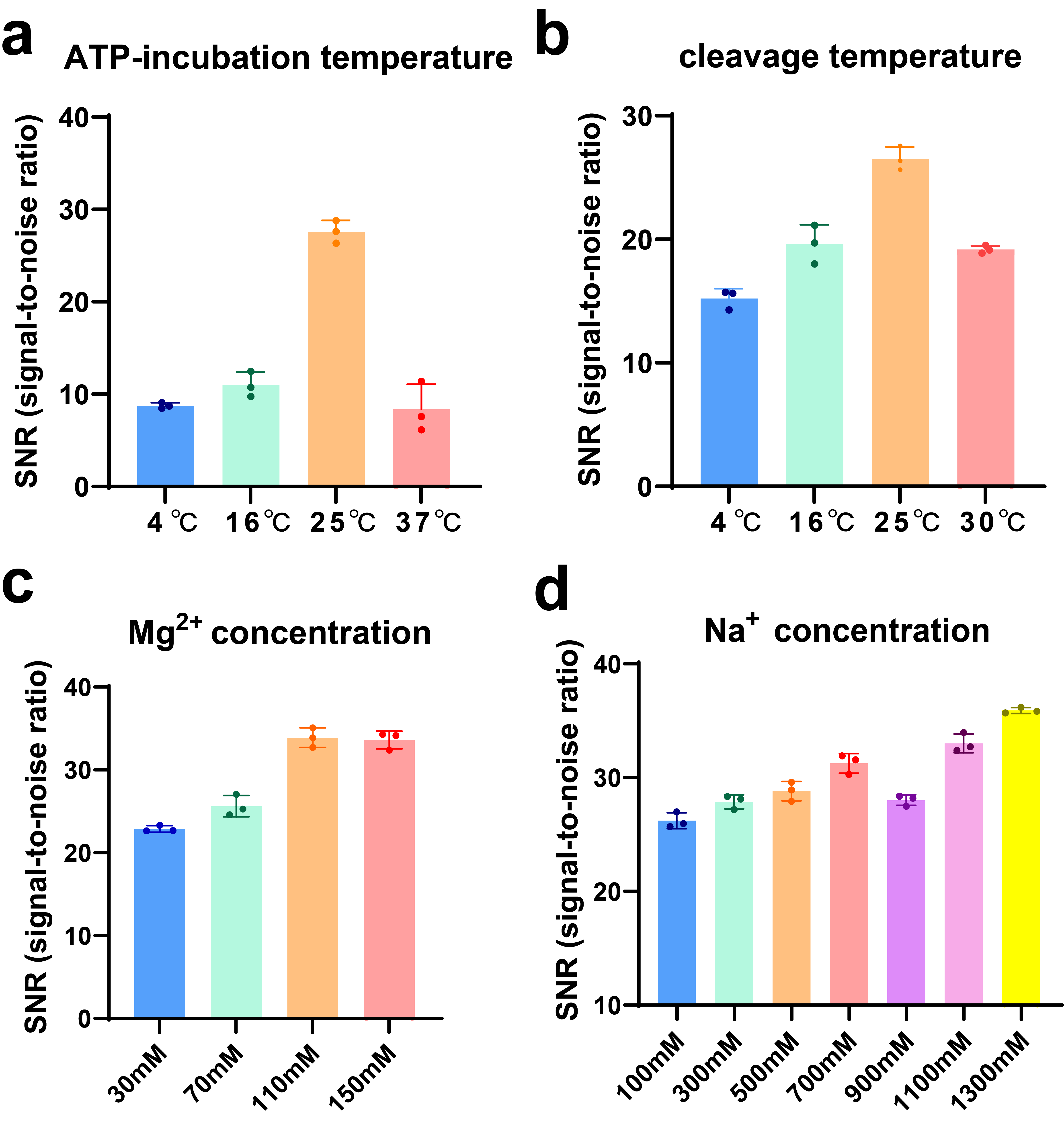
**

**Figure S7. Optimization of experimental parameters for SMART-ATP. a,** Optimization of SMART-ATP incubation temperature. **b,** Optimization of SMART cleavage temperature. **c,** Optimization of concentration of Mg^2+^ in the SMART buffer. **d,** Optimization of concentration of Na^+^ in the SMART buffer.

**
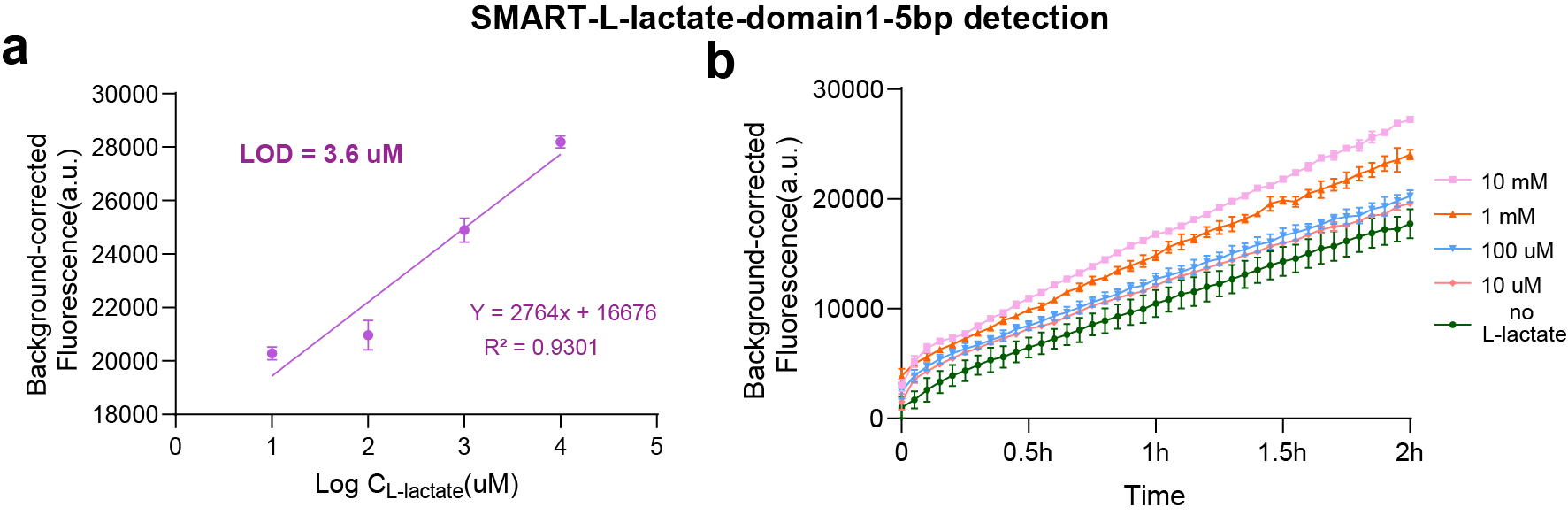
**

**Figure S8. Application of SMART platform for L-lactate detection. a,** Linear relationship of background-corrected fluorescence for different L-lactate concentrations, demonstrating good quantification capability (R^2^=0.9301). Limit of detection (LOD) is calculated to be 3.6 μM. **b,** Real-time fluorescence kinetic curves for the detection of L-lactate. SMART: 10 nM; target L-lactate: gradient diluted ranging from 10 μM to 10 mM; reporter: 500 nM; incubation time: 30 min, cleavage time: 2 hours. All experimental measurements are mean ± standard deviation (SD) with n = 3.

**
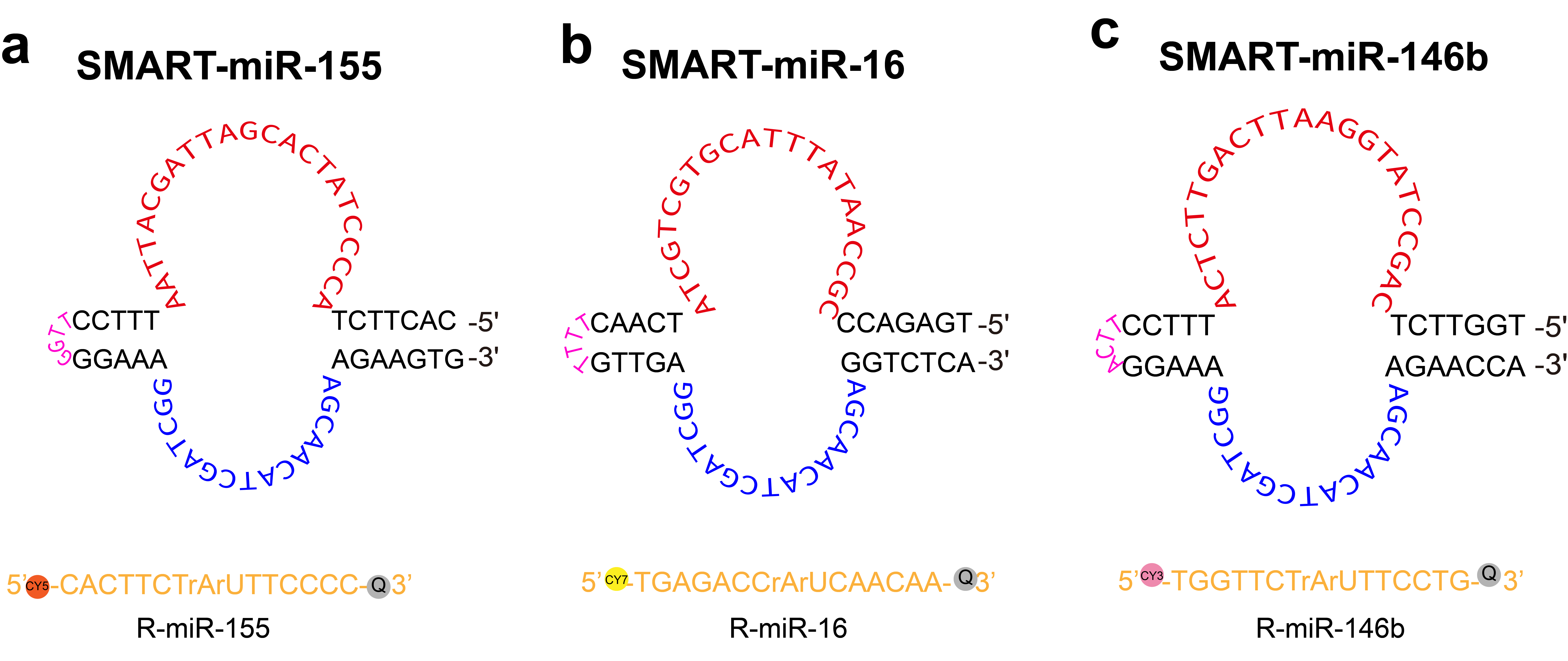
**

**Figure S9. Designs of SMART-miRNA and their corresponding RNA reporters. a-c,** Design of SMART-miRNA for detecting miR-155, miR-16, and miR-146b, respectively. The detection module is fully complementary to its miRNA target. Domain 1 is 7-bp, and domain 2 is 5-bp.

**
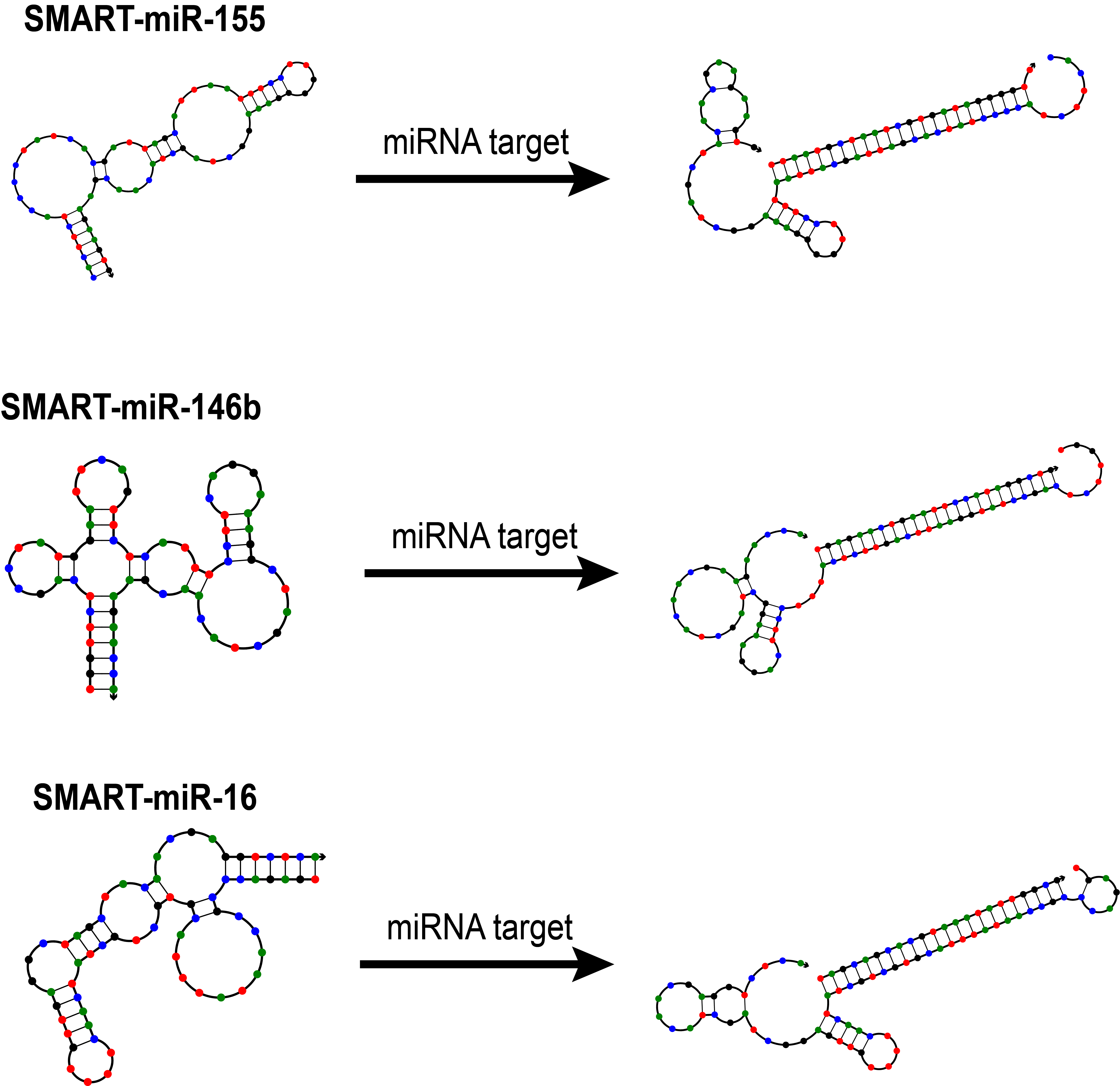
**

**Figure S10. NUPACK simulation of SMART-miRNA (7+5 bp designs) before and after miRNA binding.** Before miRNA binding, SMART-miRNA can be stably locked. After miRNA binding, domain 1 was opened for all SMART-miRNA designs.

**
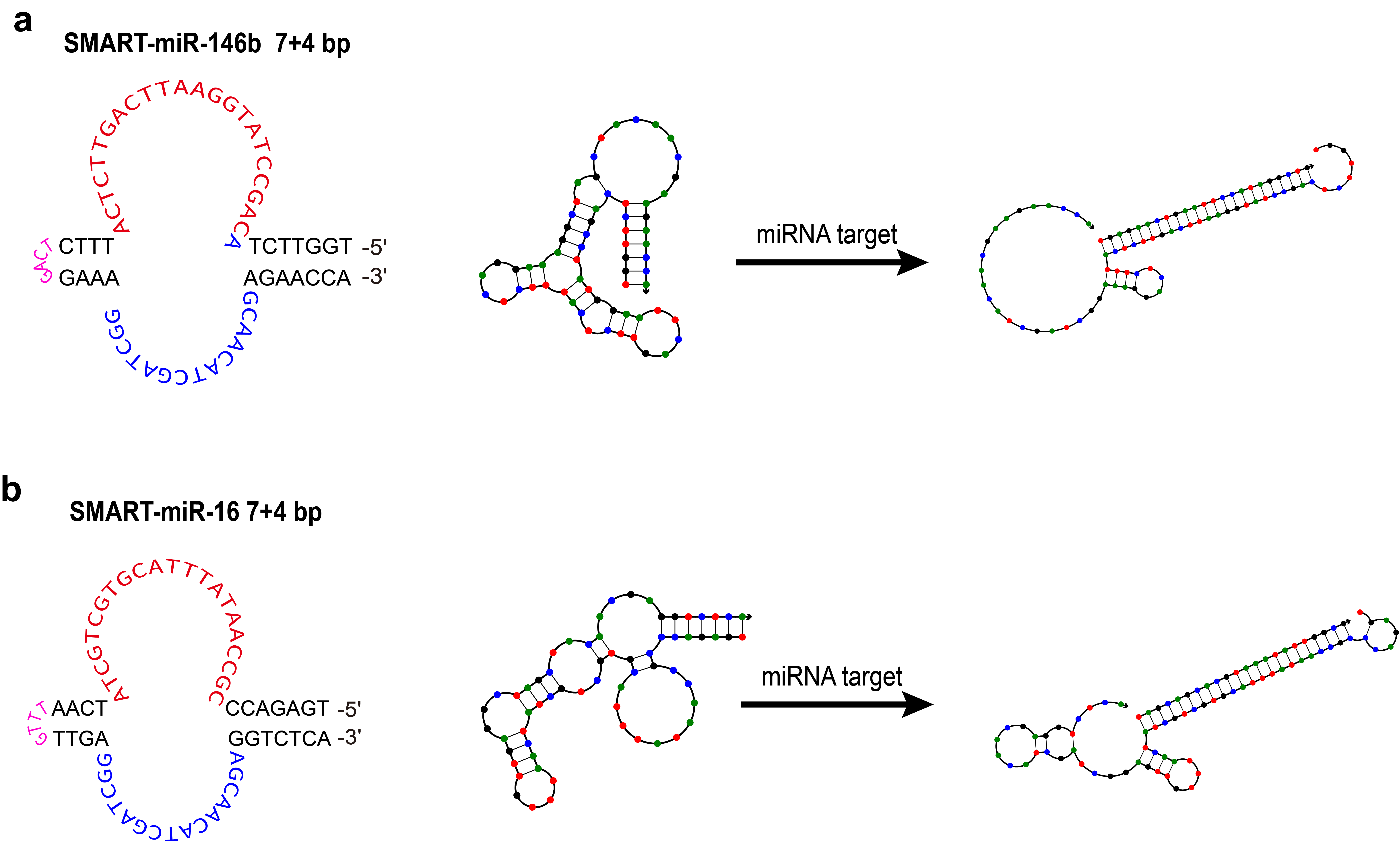
**

**Figure S11. 7+4 designs for SMART-miR-146b and SMART-miR-16.** The length of domain 2 was shortened from 5-bp to 4-bp for both designs.

**
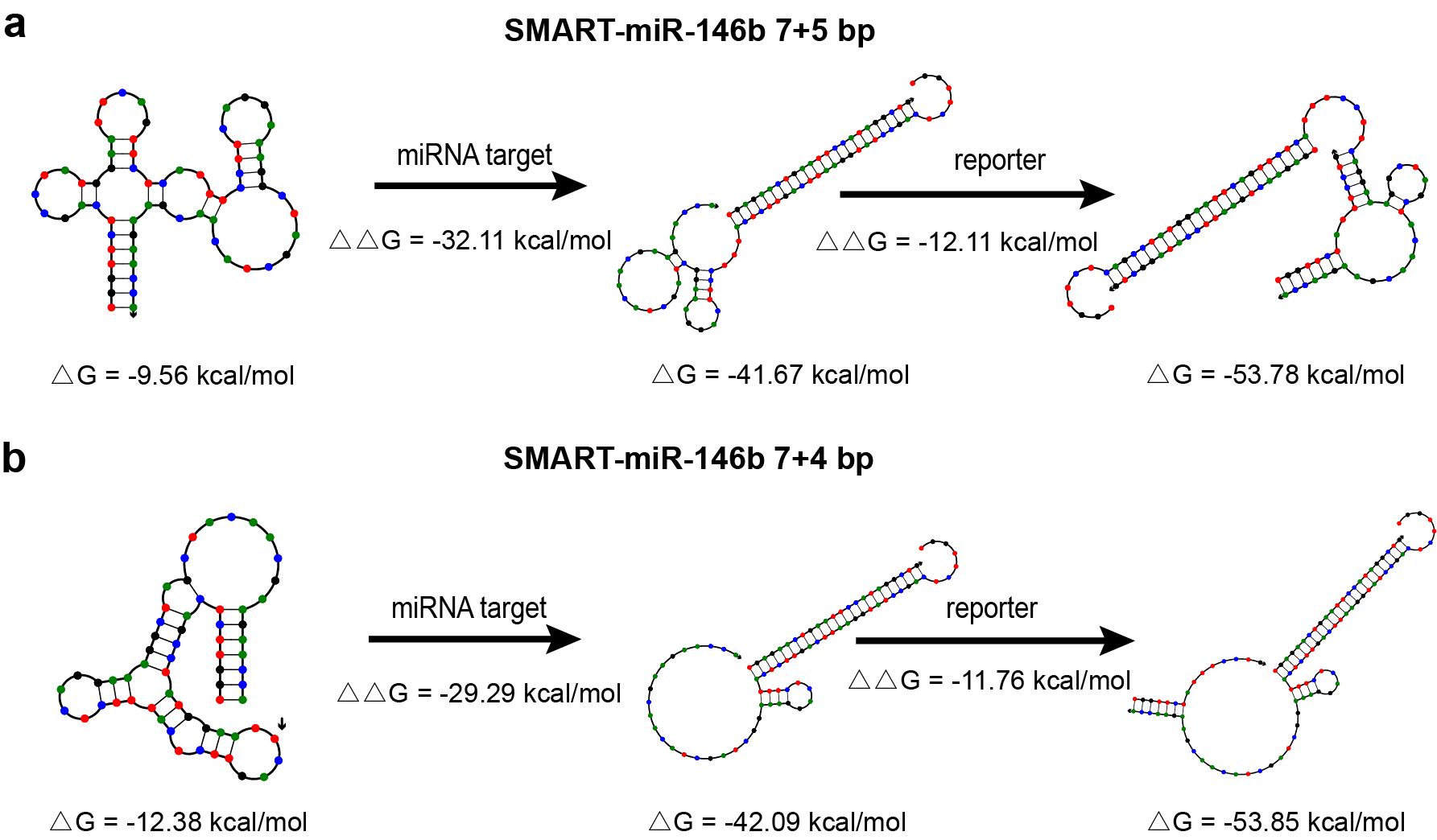
**

**Figure S12. NUPACK simulation and Gibbs free energy change of SMART-miR-146b (7+5 bp and 7+4 bp) after adding miRNA and reporter, respectively.** Ratio of SMART:target equals to 1:1. Ratio of SMART:target:reporter equals to 1:1:50. Predicted hybridization efficiencies were 80.5% for SMART-miR-146b-7+5 and 67.0% for SMART-miR-146b-7+4.

**
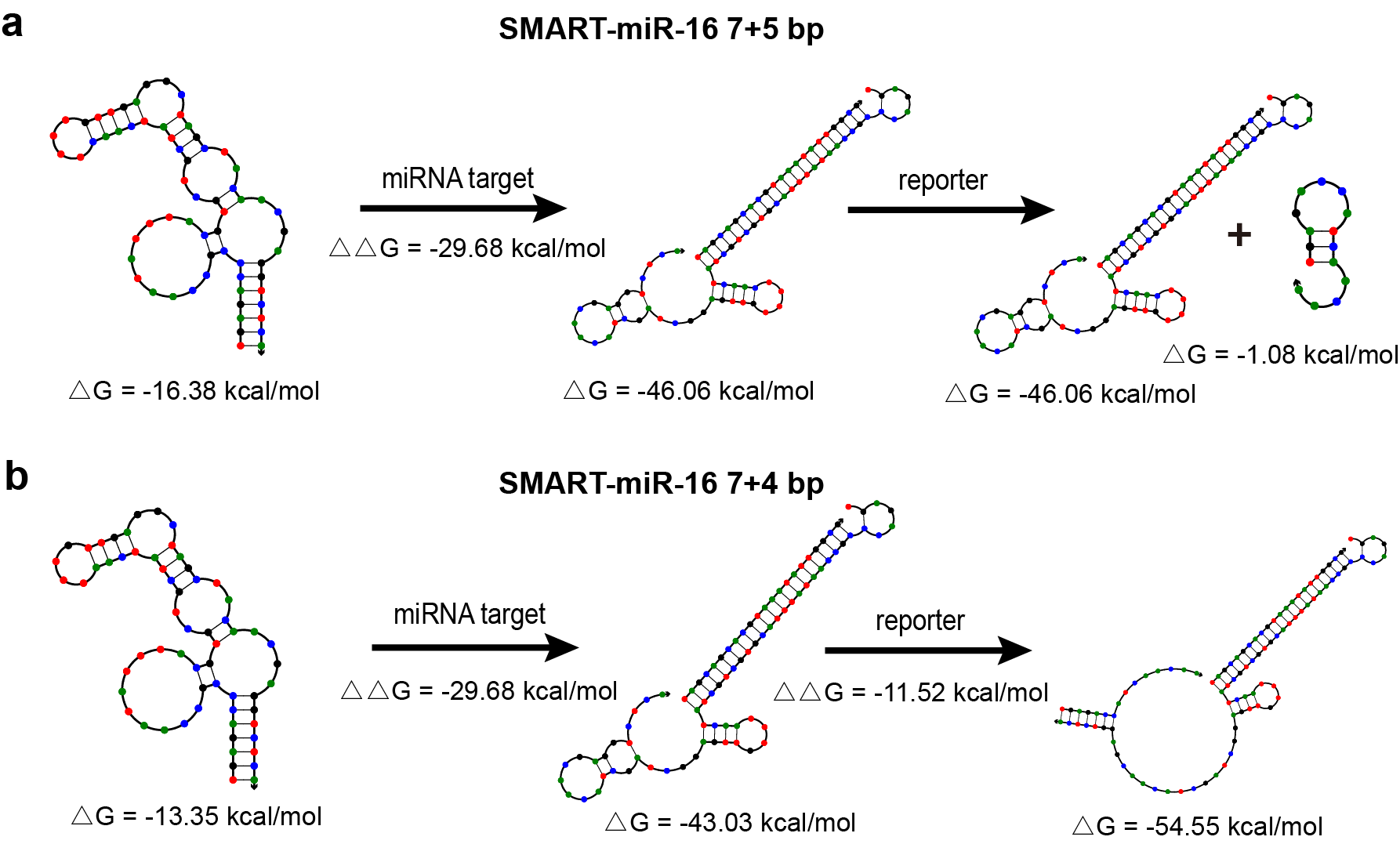
**

**Figure S13. NUPACK simulation and Gibbs free energy change of SMART-miR-16 (7+5 bp and 7+4 bp design) after adding miRNA and reporter, respectively.** Ratio of SMART:target equals to 1:1. Ratio of SMART:target:reporter equals to 1:1:50. The predicted hybridization efficiency was 61.5% for SMART-miR-16-7+4, while the SMART-miR-16-7+5 design showed no predicted hybridization with the reporter.

**
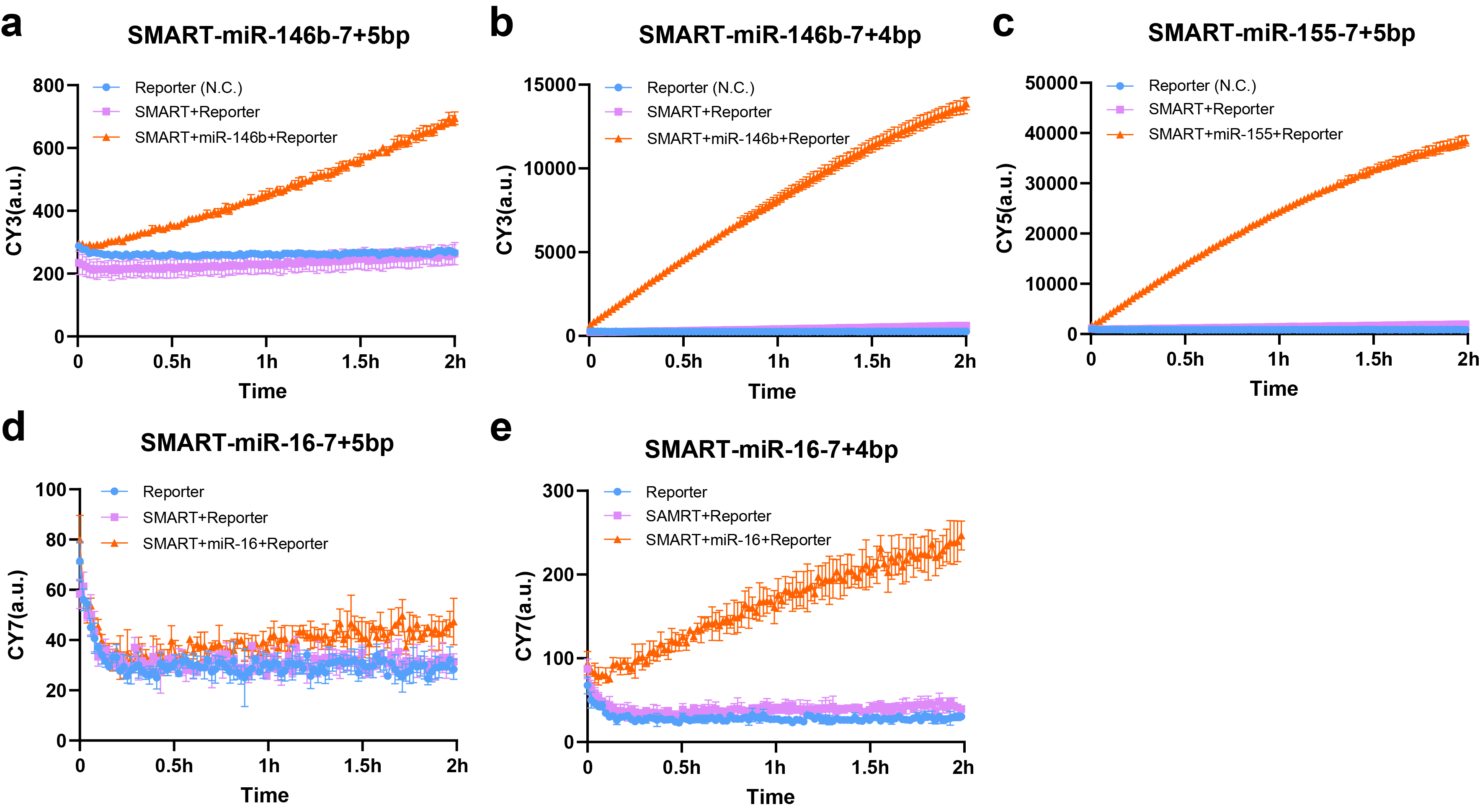
**

**Figure S14. Screening SMART-miRNA designs. a-e**, Systematic optimization of base-pair length in domain 2 (4 to 5-bp) of the SMART-miRNA for miRNA detection. The reaction mixture contained 10 nM SMART-miRNA and 10 nM target miRNA. Time-dependent cleavage of diverse sequence complementary substrates over 2 hours. All experimental measurements are mean ± standard deviation (SD) with n = 3. The initial decrease in fluorescence observed in the kinetic traces is attributed to transient non-equilibrium effects at the start of measurement, including incomplete mixing-induced concentration heterogeneity and temperature equilibration of the microplate reader, which can temporarily influence the signal. This effect is more pronounced at low fluorescence intensities (e.g., Cy7-based detection of miR-16), where small fluctuations are relatively amplified, whereas at higher signal levels such variations are masked and the subsequent signal increase reflects the true reaction kinetics.

**
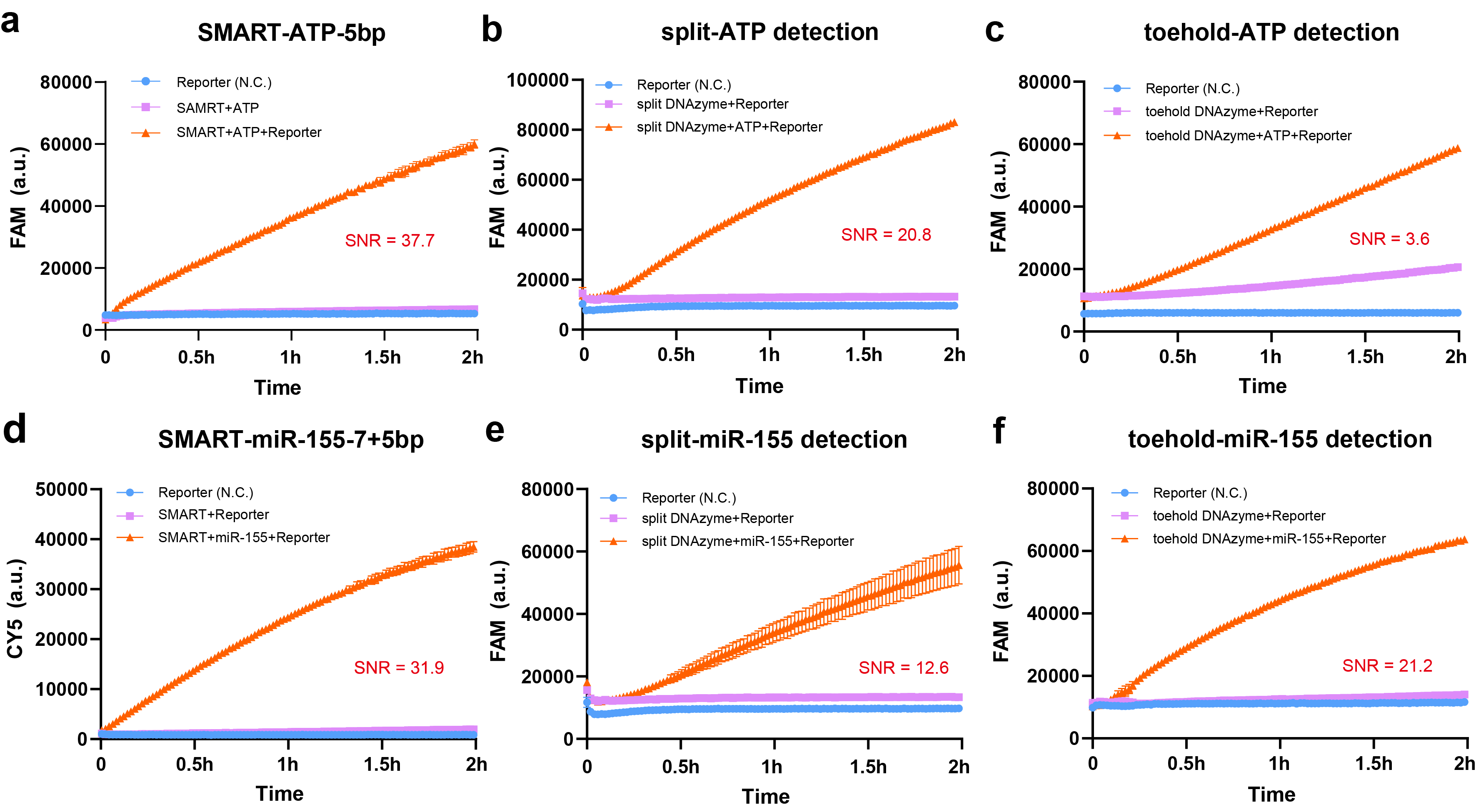
**

**Figure S15. Comparison of SMART with conventional split-and-resume and toehold-mediated DNAzyme strategies. a-c,** Real-time fluorescence kinetic curves for ATP detection using different strategies: **a,** SMART strategy; **b,** split-and-resume strategy; **c,** toehold-mediated strategy. All contained 10 nM allosteric DNAzyme, 100 μM ATP, and 500 nM reporter. **d-f,** Real-time fluorescence kinetic curves for the detection of miR-155 using different strategies: **d,** SMART strategy; **e,** split-and-resume strategy; **f,** toehold-mediated strategy. All contained 10 nM allosteric DNAzyme, 10 nM miR-155, and 500 nM reporter. Note that panel (d) displays CY5 fluorescence intensity, whereas panels (a–c, e, f) display FAM fluorescence intensity. To take rational comparison, we adopted the best sequence design and detection conditions published in the respective papers. All experimental measurements are mean ± standard deviation (SD) with n = 3.

**
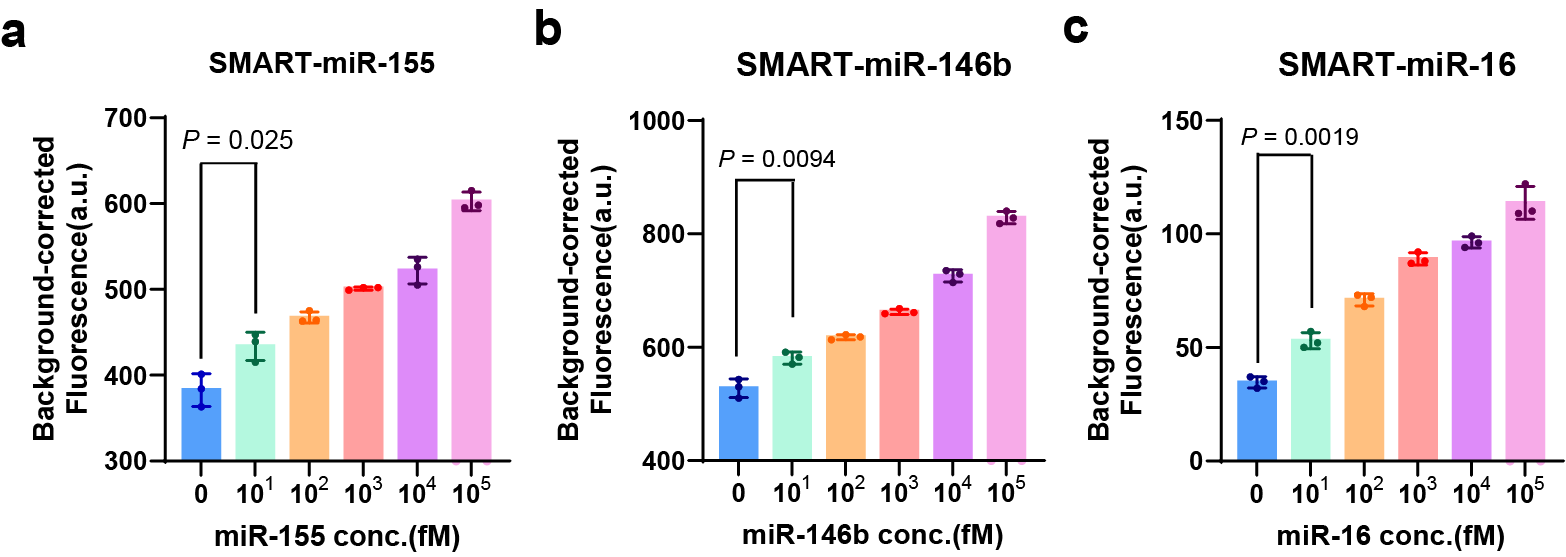
**

**Figure S16. Detection performance of SMART-miRNA with a series of miRNA concentrations. a,** Background-corrected fluorescence generated by SMART-miR-155 after incubation with different concentrations of miR-155. **b,** Background-corrected fluorescence generated by SMART-miR-146b after incubation with different concentrations of miR-146b. **c,** Background-corrected fluorescence generated by SMART-miR-16 after incubation with different concentrations of miR-16. All experiments were conducted under such a condition: SMART: 10 nM; target miRNAs: gradient diluted ranging from 10 fM to 100 pM; reporter: 500 nM; incubation time: 30 min, cleavage time: 2 hours. All experimental measurements are mean ± standard deviation (SD) with n = 3.

**
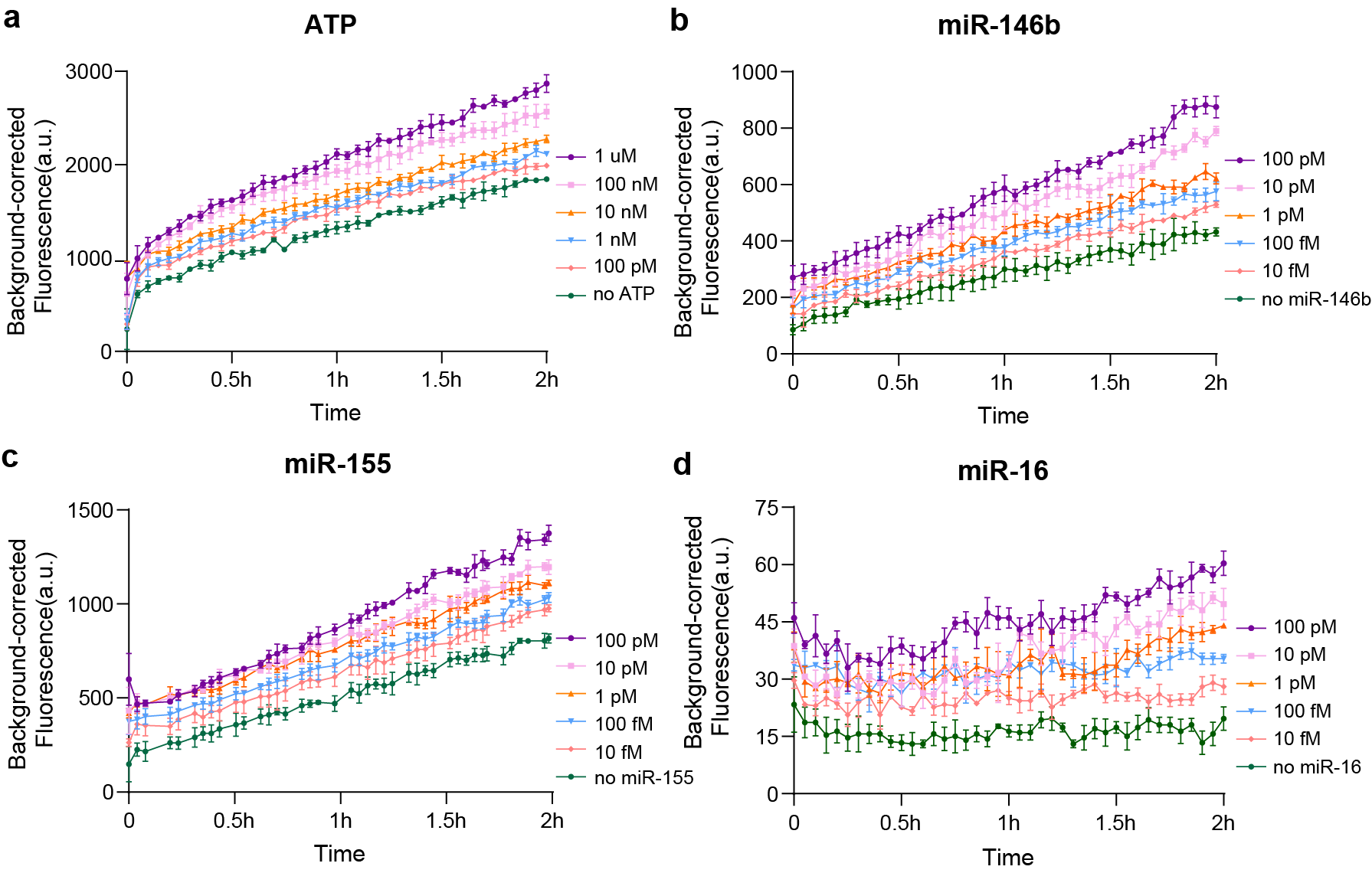
**

**Figure S17. Detection performance of SMART-ATP and SMART-miRNA systems: kinetic responses across a series of target concentrations. a,** Background-corrected fluorescence generated by SMART-ATP after incubation with different concentrations of ATP. **b,** Background-corrected fluorescence generated by SMART-miR-146b after incubation with different concentrations of miR-146b. **c,** Background-corrected fluorescence generated by SMART-miR-155 after incubation with different concentrations of miR-155. **d,** Background-corrected fluorescence generated by SMART-miR-16 after incubation with different concentrations of miR-16. All experiments were conducted under such a condition: SMART: 10 nM; target ATP: gradient diluted ranging from 100 pM to 1 μM; target miRNAs: gradient diluted ranging from 10 fM to 100 pM; reporter: 500 nM; incubation time: 30 min, cleavage time: 2 hours. All experimental measurements are mean ± standard deviation (SD) with n = 3. The initial decrease in fluorescence observed in the kinetic traces is attributed to transient non-equilibrium effects at the start of measurement, including incomplete mixing-induced concentration heterogeneity and temperature equilibration of the microplate reader, which can temporarily influence the signal. This effect is more pronounced at low fluorescence intensities (e.g., Cy5/Cy7-based detection of miR-155 and miR-16), where small fluctuations are relatively amplified, whereas at higher signal levels such variations are masked and the subsequent signal increase reflects the true reaction kinetics.

**

**

**Figure S18. Examination of the detection specificity of SMART targeting miR-146b.** miRNAs of highly homologous family members (miR-146a) or single-nucleotide mismatches of miR-146b were detected and compared to the wild-type miR-146b target. Background-corrected fluorescence was normalized by setting the value for miR-146b as 100%. SMART: 10 nM; target: 10 nM; reporter: 500 nM; reaction time: 2 hours. All experimental measurements are mean ± standard deviation (SD) with n = 3.

**
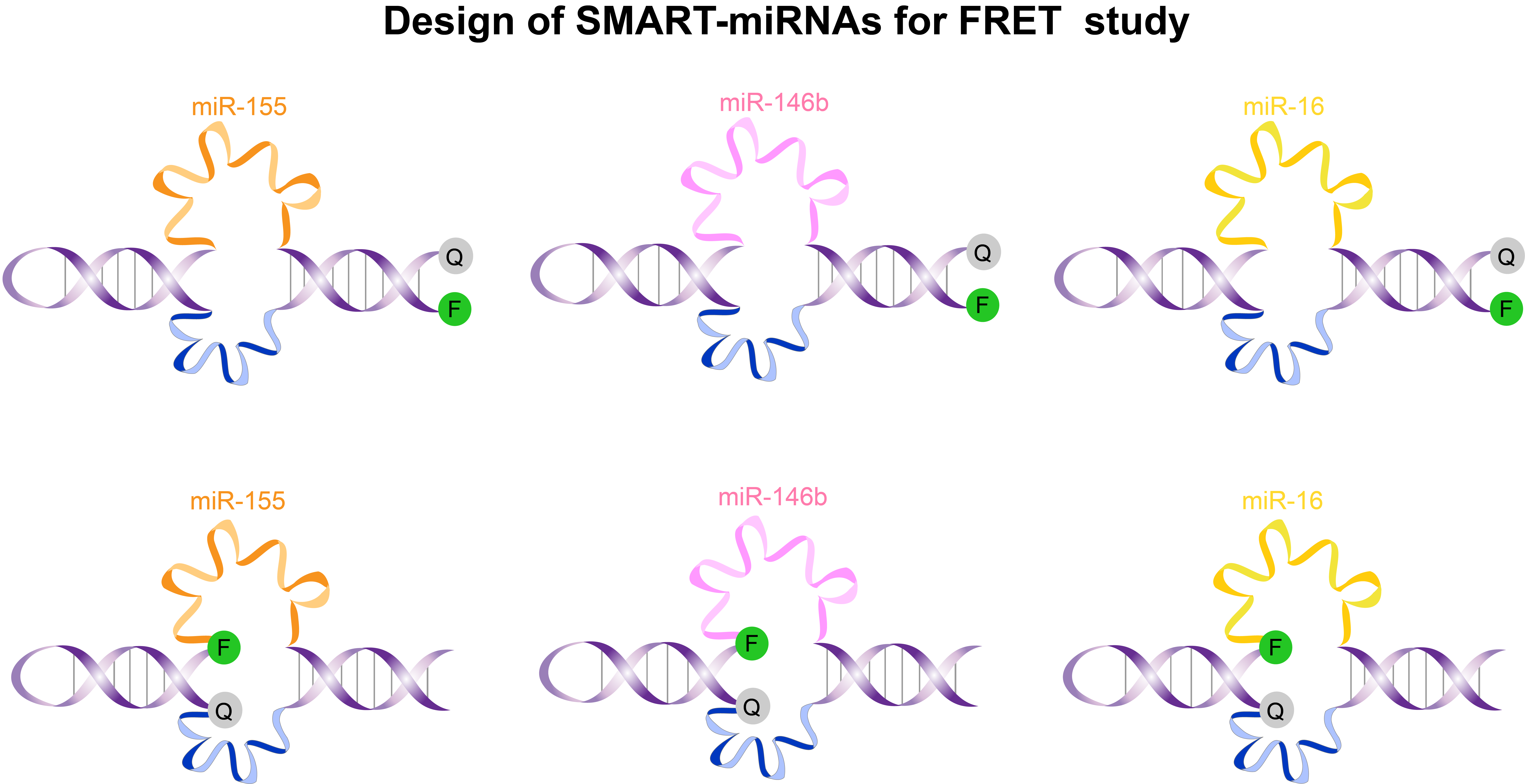
**

**Figure S19. Designs of SMART-miRNA for the FRET study.** FAM fluorophore-quencher pair labeled at terminal of domain 1 or at junctional sites between domain 2, detection module, and catalytic module.

**
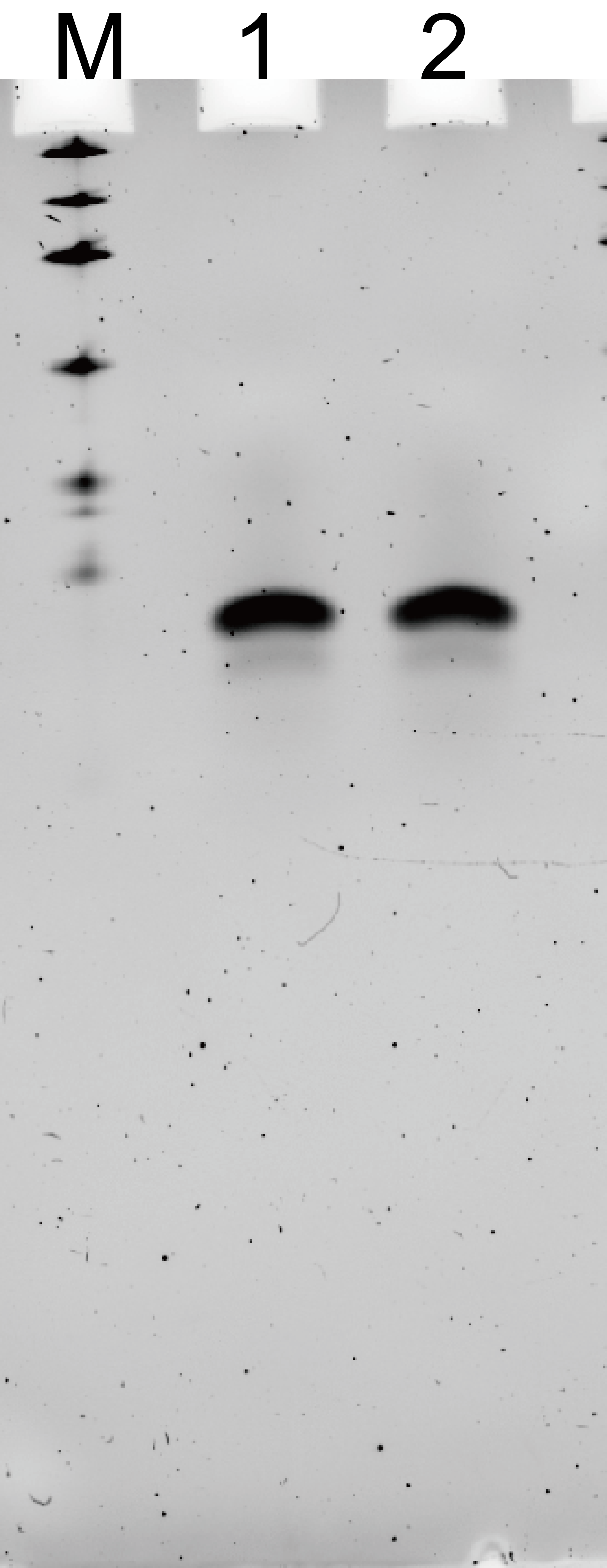
**

50bp

**Figure S20. Denaturing PAGE for miRNA integrity after heat treatment.** Lane M: single-strand RNA ladder; Lane 1: 2 μM synthetic miRNA in urine sample after 70 °C, 5min treatment. Lane 2: unheated control. ImageJ densitometry showed 98.34% miRNA retention (band intensity: 494795 vs. 503168), indicating high miRNA integrity.

**
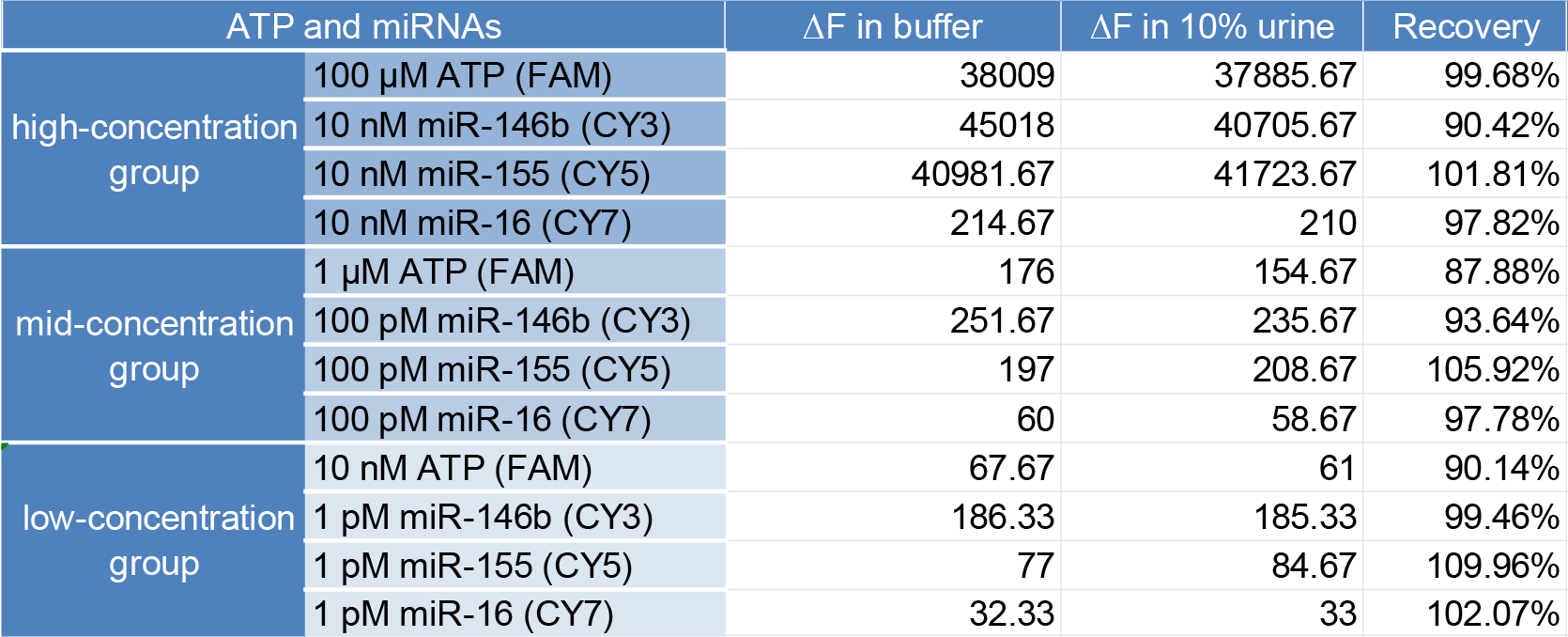
**

**Figure S21. Recovery test for detection of four targets (ATP, miR-146b, miR-155 and miR-16) in 10% urine samples.** High-concentration group contained 100 μM ATP and 10 nM miRNAs each; mid-concentration group contained 1 μM ATP and 100 pM miRNAs each; low-concentration group contained 10 nM ATP and 1 pM miRNAs each. Test samples contained targets spiked in buffer with 10% urine; controls contained targets in buffer alone. ΔF was measured fluorescence subtracting the corresponding blank fluorescence, and recovery (%) = (ΔF in 10% urine / ΔF in buffer) × 100%. The recovery efficacy was ranged from 87% to 110%.

**Table S1. Oligonucleotides used in all SMART assays.**

| **Name** | **Sequence (from 5'-end to 3'-end)** |
| --- | --- |
| SMART-ATP-8+8 | GCACTTCTACCTGGGGGAGTATTGCGGAGGAAGGTTTTCCCCTTTTTAGGGGAAAGGCTAGCTACAACGA AGAAGTGC |
| SMART-ATP-8+7 | GCACTTCTACCTGGGGGAGTATTGCGGAGGAAGGTTTTCCCCTTTTGGGGAAAGGCTAGCTACAACGAAGAAGTGC |
| SMART-ATP-8+6 | GCACTTCTACCTGGGGGAGTATTGCGGAGGAAGGTTTTCCCTTTGGGGAAAGGCTAGCTACAACGAAGAAGTGC |
| SMART-ATP-8+5 | GCACTTCTACCTGGGGGAGTATTGCGGAGGAAGGTTTTCCTTGGGGAAAGGCTAGCTACAACGAAGAAGTGC |
| SMART-ATP-8+4 | GCACTTCTACCTGGGGGAGTATTGCGGAGGAAGGTTTTCTGGGGAAAGGCTAGCTACAACGAAGAAGTGC |
| SMART-ATP-8+3 | GCACTTCTACCTGGGGGAGTATTGCGGAGGAAGGTTTTGGGGAAAGGCTAGCTACAACGAAGAAGTGC |
| SMART-ATP-7+8 | CACTTCTACCTGGGGGAGTATTGCGGAGGAAGGT TTTCCCCTTTTTAGGGGAAAGGCTAGCTACAACGAAGAAGTG |
| SMART-ATP-7+7 | CACTTCTACCTGGGGGAGTATTGCGGAGGAAGGT TTTCCCCTTTTGGGGAAA GGCTAGCTACAACGAAGAAGTG |
| SMART-ATP-7+6 | CACTTCTACCTGGGGGAGTATTGCGGAGGAAGGTTTTCCCTTTGGGGAAA GGCTAGCTACAACGAAGAAGTG |
| SMART-ATP-7+5 | CACTTCTACCTGGGGGAGTATTGCGGAGGAAGGT TTTCCTTGGGGAAAGGCTAGCTACAACGAAGAAGTG |
| SMART-ATP-7+4 | CACTTCTACCTGGGGGAGTATTGCGGAGGAAGGTTTTCTGGGGAAAGGCTAGCTACAACGA AGAAGTG |
| SMART-ATP-7+3 | CACTTCTACCTGGGGGAGTATTGCGGAGGAAGGTTTTGGGGAAAGGCTAGCTACAACGAAGAAGTG |
| SMART-ATP-6+8 | ACTTCTACCTGGGGGAGTATTGCGGAGGAAGGTTTTCCCCTTTTTAGGGGAAAGGCTAGCTACAACGAAGAAGT |
| SMART-ATP-6+7 | ACTTCTACCTGGGGGAGTATTGCGGAGGAAGGTTTTCCCCTTTTGGGGAAAGGCTAGCTACAACGAAGAAGT |
| SMART-ATP-6+6 | ACTTCTACCTGGGGGAGTATTGCGGAGGAAGGTTTTCCCTTTGGGGAAAGGCTAGCTACAACGAAGAAGT |
| SMART-ATP-6+5 | ACTTCTACCTGGGGGAGTATTGCGGAGGAAGGTTTTCCTTGGGGAAAGGCTAGCTACAACGAAGAAGT |
| SMART-ATP-6+4 | ACTTCTACCTGGGGGAGTATTGCGGAGGAAGGTTTTCTGGGGAAAGGCTAGCTACAACGAAGAAGT |
| SMART-ATP-6+3 | ACTTCTACCTGGGGGAGTATTGCGGAGGAAGGTTTTGGGGAAAGGCTAGCTACAACGAAGAAGT |
| SMART-ATP-5+8 | CTTCTACCTGGGGGAGTATTGCGGAGGAAGGTTTTCCCCTTTTTAGGGGAAAGGCTAGCTACAACGAAGAAG |
| SMART-ATP-5+7 | CTTCTACCTGGGGGAGTATTGCGGAGGAAGGTTTTCCCCTTTTGGGGAAAGGCTAGCTACAACGAAGAAG |
| SMART-ATP-5+6 | CTTCTACCTGGGGGAGTATTGCGGAGGAAGGTTTTCCCTTTGGGGAAAGGCTAGCTACAACGAAGAAG |
| SMART-ATP-5+5 | CTTCTACCTGGGGGAGTATTGCGGAGGAAGGTTTTCCTTGGGGAAAGGCTAGCTACAACGAAGAAG |
| SMART-ATP-5+4 | CTTCTACCTGGGGGAGTATTGCGGAGGAAGGTTTTCTGGGGAAAGGCTAGCTACAACGAAGAAG |
| SMART-ATP-5+3 | CTTCTACCTGGGGGAGTATTGCGGAGGAAGGTTTTGGGGAAAGGCTAGCTACAACGAAGAAG |
| SMART-ATP-4bp-insertion | TATACCTGGGGGAGTATTGCGGAGGAAGGTTTTCCTTGGGGAAAGGCTAGCTACAACGAAGGTATA |
| SMART-ATP-5bp-insertion | AAACCTGGGGGAGTATTGCGGAGGAAGGTTTTCCTTGGGGAAAGGCTAGCTACAACGACAGGTTT |
| SMART-ATP-6bp-insertion | TACCTGGGGGAGTATTGCGGAGGAAGGTTTTCCTTGGGGAAAGGCTAGCTACAACGACCAGGTA |
| SMART-ATP-7bp-insertion | ACCTGGGGGAGTATTGCGGAGGAAGGTTTTCCTTGGGGAAAGGCTAGCTACAACGACCCAGGT |
| SMART-ATP-domain2-5bp-insertion | AAACCTGACCTGGGGGAGTATTGCGGAGGAAGGTTTGGACCTTAGGCTAGCTACAACGACAGGTTT |
| R-ATP | /FAM/CACTTCT rArU TTCCCC/BHQ1/ |
| R-ATP-4bp | /FAM/TATACCT rArU TTCCCC/BHQ1/ |
| R-ATP-5bp | /FAM/AAACCTG rArU TTCCCC/BHQ1/ |
| R-ATP-6bp | /FAM/TACCTGG rArU TTCCCC/BHQ1/ |
| R-ATP-7bp | /FAM/ACCTGGG rArU TTCCCC/BHQ1/ |
| R-ATP-domain2-5bp | /FAM/AAACCTG rArU AAGGTC/BHQ1/ |
| SMART-ATP-FAM-end | /FAM/AAACCTGGGGGAGTATTGCGGAGGAAGGTTTTCCTTGGGGAAAGGCTAGCTACAACGA CAGGTTT /BHQ1/ |
| SMART-ATP-FAM-medium | AAACCTGGGGGAGTATTGCGGAGGAAGGT/i6FAM/TTTCCTTGGGGAAA /iBHQ1/ GGCTAGCTACAACGACAGGTTT |
| R*-ATP-DNA | AAACCTGATTTCCCC |
| SMART-miR-146b-5p-FAM-end | /FAM/TGGTTCTCAGCCTATGGAATTCAGTTCTCATTTCTCAGGAAAGGCTAGCTACAACGAAGAACCA /BHQ1/ |
| SMART-miR-146b-5p-FAM-medium | TGGTTCTCAGCCTATGGAATTCAGTTCTCA/i6FAM/TTTCTCAGGAAA/iBHQ1/GGCTAGCTACAACGAAGAACCA |
| R*-miR-146b-5p-DNA | TGGTTCTATTTCCTG |
| SMART-miR-155-5p-FAM-end | /FAM/CACTTCTACCCCTATCACGATTAGCATTAATTTCCTTGGGGAAAGGCTAGCTACAACGAAGAAGTG/BHQ1/ |
| SMART-miR-155-5p-FAM-medium | CACTTCTACCCCTATCACGATTAGCATTAA/i6FAM/TTTCCTTGGGGAAA/iBHQ1/GGCTAGCTACAACGAAGAAGTG |
| R*-miR-155-5p-DNA | CACTTCTATTTCCCC |
| SMART-miR-16-5p-FAM-end | /FAM/TGAGACCCGCCAATATTTACGTGCTGCTATCAATTTGTTGAGGCTAGCTACAACGAGGTCTCA/BHQ1/ |
| SMART-miR-16-5p-FAM-medium | TGAGACCCGCCAATATTTACGTGCTGCTA/i6FAM/TCAATTTGTTGA/iBHQ1/GGCTAGCTACAACGAGGTCTCA |
| R*-miR-16-5p-DNA | TGAGACCATCAACAA |
| HSA-miR-146b-5p | UGAGAACUGAAUUCCAUAGGCUG |
| HSA-miR-155-5p | UUAAUGCUAAUCGUGAUAGGGGUU |
| HSA-miR-16-5p | UAGCAGCACGUAAAUAUUGGCG |
| SMART-miR-146b-7+5 | TGGTTCTCAGCCTATGGAATTCAGTTCTCATTTCCTTCAGGAAAGGCTAGCTACAACGAAGAACCA |
| SMART-miR-146b-7+4 | TGGTTCTCAGCCTATGGAATTCAGTTCTCATTTCTCAGGAAAGGCTAGCTACAACGAAGAACCA |
| SMART-miR-155-7+5 | CACTTCTACCCCTATCACGATTAGCATTAATTTCCTTGGGGAAAGGCTAGCTACAACGA AGAAGTG |
| SMART-miR-16-7+5 | TGAGACCCGCCAATATTTACGTGCTGCTATCAACTTTTGTTGAGGCTAGCTACAACGAGGTCTCA |
| SMART-miR-16-7+4 | TGAGACCCGCCAATATTTACGTGCTGCTATCAATTTGTTGAGGCTAGCTACAACGAGGTCTCA |
| R-miR-146b-5p | /cy3/TGGTTCTrArUTTCCTG/BHQ2/ |
| R-miR-155-5p | /cy5/CACTTCT rArU TTCCCC/BHQ2/ |
| R-miR-16-5p | /cy7/TGAGACC rArU CAACAA/BHQ3/ |

**Table S2. Oligonucleotides used in comparison with traditional allosteric DNAzyme**

| **Name** | **Sequence (from 5'-end to 3'-end)** |
| --- | --- |
| ATP-Output-DNAzyme | TGAGATATCTCCGAGCCGGACGAATACTCTTG |
| ATP-Complementary-DNAzyme | GGCTCGGAGATATCTCATTTGCAACTTAG |
| DNAzyme-ATP-toehold | TAAGTTGCTTCTTGGGGGAGTATTGCGGAGGAAAGTTATGAGATATCTCCGAGCC |
| substrate-ATP-toehold | /BHQ1/CAAGAGTATrAGGATATCTC/FAM/ |
| DNAzyme-toehold-miR-155 | AATCGTGATAGGGGTTTCTCTTCTCCGAGCCGGTCGAAATAGT |
| lock6-miR-155 | AAGAGAAACCCCTATCACGATTAGCATTAA |
| HSA-miR-155-5p | UUAAUGCUAAUCGUGAUAGGGGUU |
| substrate-miR-155-toehold-/FAM/ | /FAM/CACTATrAGGAAGAGAT/BHQ1/ |
| split-DNAzyme-ATP | TGCCCAGGGTCCGAGCCGGGGGAGTATTGCGGAGGAGCGGGGGAGTATTGCGGAGGAGCGGTCGAAGCGGAAACCTT |
| FAM-split-reporter | /FAM/AAGGTTTCCGCrAGCCCTGGGCA/BHQ1/ |
| split-DNAzyme-miR-155 | TGCCCAGGGTCCGAGCGATTAGCATTAATTTTTTTTTTTTTTTTTTTTTTTTTTTTTTTTTTTTTTTTTTTTTTTTTTTTTTTTTTTAACCCCTATCAC CGGTCGAAGCGGAAACCTT |

**Table S3. Information of patients.**

| SMART for UTI diagnosis assay | | | | | |
| --- | --- | --- | --- | --- | --- |
| No. | sex | age | clinical diagnose | infection type (culture results) | Urine System diseases (clinical diagnosis) |
| UTI-1 | male | 73 | (+) | *Escherichia coli* | *UTI, Cystitis* |
| UTI-2 | female | 79 | (+) | *Escherichia coli* | *cUTI* |
| UTI-3 | female | 74 | (+) | *Escherichia coli* | *CDK* |
| UTI-4 | male | 46 | (+) | *Escherichia coli* | *UTI, BPH* |
| UTI-5 | female | 31 | (+) | *Escherichia coli* | *UTI* |
| UTI-6 | female | 71 | (+) | *Actinomyces turicensis* | *cUTI* |
| UTI-7 | female | 72 | (+) | *Candida glabrata* | *UTI* |
| UTI-8 | female | 75 | (+) | *Klebsiella pneumoniae* | *UTI* |
| UTI-9 | female | 85 | (+) | *Candida tropicalis* | *UTI* |
| UTI-10 | male | 46 | (+) | *Enterococcus faecium* | *AKI* |
| UTI-11 | male | 82 | (+) | *Enterococcus faecalis* | *UTI* |
| UTI-12 | male | 72 | (+) | *Enterococcus faecalis* | *UTI* |
| UTI-13 | male | 67 | (+) | *Lactobacillus* | *UTI, BPH* |
| UTI-14 | female | 51 | (+) | *Lactobacillus* | *UTI* |
| UTI-15 | female | 26 | (+) | *Lactobacillus jensenii* | *UTI* |
| UTI-16 | female | 54 | (+) | *Enterobacter cloacae* | *Ureteral stone with hydronephrosis and infection* |
| UTI-17 | male | 76 | (+) | *Streptococcus agalactiae* | *UTI, BPH* |
| UTI-18 | male | 64 | (+) | *Enterobacter hormaechei* | *UTI* |
| UTI-19 | female | 75 | (+) | *Enterococcus faecalis* | *cUTI* |
| UTI-20 | female | 54 | (+) | *Escherichia coli* | *UTI, Hematuria* |
| UTI-21 | female | 65 | (+) | *Escherichia coli* | *Ureteral stone with hydronephrosis and infection* |
| UTI-22 | female | 62 | (+) | *Escherichia coli* | *UTI, Renal tumor* |
| UTI-23 | male | 83 | (+) | *Enterococcus faecium* | *UTI, BPH, BC* |
| UTI-24 | female | 52 | (+) | *Corynebacterium glucuronolyticum* | *UTI* |
| UTI-25 | male | 79 | (+) | *Staphylococcus aureus* | *UTI* |
| UTI-26 | female | 88 | (+) | *Pseudomonas aeruginosa* | *UTI* |
| UTI-27 | male | 80 | (+) | *Escherichia coli* | *UTI, BPH* |
| UTI-28 | female | 92 | (+) | *Escherichia coli* | *cUTI* |
| UTI-29 | male | 65 | (+) | *Proteus mirabilis* | *UTI* |
| UTI-30 | female | 78 | (+) | *Candida tropicalis* | *UTI* |
| UTI-31 | female | 43 | (+) | *Streptococcus agalactiae* | *BC* |
| UTI-32 | female | 54 | (+) | *Enterococcus faecalis* | *Ureteral stricture, Hydronephrosis with renal calculi* |
| UTI-33 | male | 68 | (+) | *Escherichia coli* | *BPH* |
| UTI-34 | female | 54 | (+) | *Enterococcus faecalis* | *UTI, Ureteral stone* |
| UTI-35 | male | 43 | (+) | *Staphylococcus warneri* | *Nephrolithiasis* |
| UTI-36 | male | 77 | (+) | *Aerococcus urinae* | *UTI, BPH* |
| UTI-37 | male | 75 | (+) | *Enterococcus faecium* | *BPH* |
| UTI-38 | male | 44 | (+) | *Chlamydia trachomatis & Neisseria gonorrhoeae* | *UTI* |
| UTI-39 | female | 37 | (+) | *Enterococcus faecium* | *UTI* |
| UTI-40 | male | 41 | (+) | *Pseudomonas aeruginosa* | *UTI* |
| UTI-41 | female | 59 | (+) | *Proteus mirabilis* | *UTI, Renal stone with hydronephrosis and infection* |
| UTI-42 | female | 80 | (+) | *Escherichia coli* | *BC* |
| UTI-43 | female | 70 | (+) | *Escherichia coli* | *UTI* |
| UTI-44 | female | 30 | (+) | *Escherichia coli* | *cUTI* |
| UTI-45 | male | 43 | (+) | *Enterococcus faecium* | *cUTI* |
| UTI-46 | female | 72 | (+) | *Staphylococcus epidermidis* | *UTI* |
| UTI-47 | female | 60 | (+) | *Escherichia coli* | *UTI* |
| UTI-48 | male | 60 | (+) | *Morganella morganii subsp. sibonii* | *UTI, BPH* |
| UTI-49 | female | 49 | (+) | *Escherichia coli* | *Renal stone with hydronephrosis and infection* |
| UTI-50 | male | 79 | (+) | *Enterococcus faecalis* | *cUTI, BPH* |
| UTI-51 | male | 72 | (+) | *Streptococcus agalactiae* | *UTI, BPH* |
| UTI-52 | male | 21 | (+) | *Corynebacterium striatum* | *Ureteral stone with hydronephrosis and infection* |
| UTI-53 | male | 73 | (+) | *Citrobacter freundii* | *UTI, BC* |
| UTI-54 | male | 73 | (+) | *Enterococcus faecalis & Candida albicans* | *cUTI* |
| UTI-55 | female | 56 | (+) | *Pseudomonas aeruginosa* | *UTI* |
| UTI-56 | female | 57 | (+) | *Enterococcus faecium* | *UTI* |
| UTI-57 | male | 42 | (+) | *Escherichia coli* | *UTI* |
| UTI-58 | female | 77 | (+) | *Escherichia coli* | *UTI* |
| UTI-59 | female | 35 | (+) | *Proteus mirabilis* | *UTI, BPH* |
| UTI-60 | female | 41 | (+) | *Candida tropicalis* | *UTI* |
| UTI-61 | male | 81 | (+) | *Escherichia coli* | *Hydronephrosis with renal calculi* |
| UTI-62 | male | 52 | (+) | *Escherichia coli* | *UTI* |
| UTI-63 | male | 37 | (+) | *Enterococcus faecalis* | *Ureteral stone with hydronephrosis and infection* |
| UTI-64 | female | 40 | (+) | *Escherichia coli* | *UTI* |
| UTI-65 | female | 72 | (+) | *Enterococcus faecium* | *UTI* |
| UTI-66 | male | 76 | (+) | *Escherichia coli* | *UTI* |
| H-1 | male | 54 | (-) | / | / |
| H-2 | male | 29 | (-) | / | / |
| H-3 | male | 59 | (-) | / | / |
| H-4 | female | 42 | (-) | / | / |
| H-5 | male | 27 | (-) | / | / |
| H-6 | male | 35 | (-) | / | / |
| H-7 | male | 32 | (-) | / | / |
| H-8 | male | 26 | (-) | / | / |
| H-9 | male | 34 | (-) | / | / |
| H-10 | male | 28 | (-) | / | / |
| H-11 | male | 44 | (-) | / | / |
| H-12 | male | 52 | (-) | / | / |
| H-13 | male | 31 | (-) | / | / |
| H-14 | male | 26 | (-) | / | / |
| H-15 | male | 38 | (-) | / | / |
| H-16 | female | 29 | (-) | / | / |
| H-17 | female | 39 | (-) | / | / |
| H-18 | male | 27 | (-) | / | / |
| H-19 | female | 58 | (-) | / | / |
| H-20 | female | 37 | (-) | / | / |
| H-21 | female | 37 | (-) | / | / |
| H-22 | male | 35 | (-) | / | / |
| H-23 | male | 58 | (-) | / | / |
| H-24 | female | 31 | (-) | / | / |
| H-25 | male | 35 | (-) | / | / |
| H-26 | female | 34 | (-) | / | / |
| H-27 | female | 70 | (-) | / | / |
| H-28 | female | 33 | (-) | / | / |
| H-29 | male | 36 | (-) | / | / |
| H-30 | female | 31 | (-) | / | / |
| H-31 | male | 38 | (-) | / | / |
| H-32 | female | 35 | (-) | / | / |
| H-33 | male | 29 | (-) | / | / |
| H-34 | female | 45 | (-) | / | / |
| H-35 | male | 65 | (-) | / | / |
| H-36 | male | 41 | (-) | / | / |
| H-37 | female | 59 | (-) | / | / |
| H-38 | female | 27 | (-) | / | / |
| H-39 | female | 40 | (-) | / | / |
| H-40 | female | 42 | (-) | / | / |
| H-41 | female | 31 | (-) | / | / |
| H-42 | female | 54 | (-) | / | / |
| H-43 | female | 54 | (-) | / | / |
| H-44 | female | 59 | (-) | / | / |
| H-45 | female | 58 | (-) | / | / |
| H-46 | male | 78 | (-) | / | / |
| H-47 | male | 60 | (-) | / | / |
| H-48 | male | 60 | (-) | / | / |
| H-49 | male | 29 | (-) | / | / |
| H-50 | male | 37 | (-) | / | / |
| H-51 | male | 65 | (-) | / | / |
| H-52 | male | 53 | (-) | / | / |
| H-53 | male | 33 | (-) | / | / |
| H-54 | female | 45 | (-) | / | / |
| H-55 | male | 46 | (-) | / | / |
| H-56 | female | 33 | (-) | / | / |
| H-57 | female | 36 | (-) | / | / |
| H-58 | male | 30 | (-) | / | / |
| H-59 | female | 33 | (-) | / | / |
| H-60 | female | 61 | (-) | / | / |
| H-61 | male | 39 | (-) | / | / |
| H-62 | male | 55 | (-) | / | / |
| H-63 | male | 43 | (-) | / | / |
| H-64 | female | 59 | (-) | / | / |
| H-65 | male | 70 | (-) | / | / |
| H-66 | male | 59 | (-) | / | / |
| H-67 | male | 52 | (-) | / | / |
| H-68 | male | 53 | (-) | / | / |
| H-69 | male | 37 | (-) | / | / |
| H-70 | male | 35 | (-) | / | / |
| H-71 | male | 33 | (-) | / | / |
| H-72 | male | 49 | (-) | / | / |
| H-73 | male | 34 | (-) | / | / |
| H-74 | male | 31 | (-) | / | / |
| H-75 | male | 33 | (-) | / | / |
| H-76 | male | 58 | (-) | / | / |
| H-77 | female | 50 | (-) | / | / |
| H-78 | female | 32 | (-) | / | / |
| H-79 | male | 52 | (-) | / | / |
| H-80 | male | 36 | (-) | / | / |
| H-81 | female | 25 | (-) | / | / |
| H-82 | female | 72 | (-) | / | / |
| H-83 | male | 58 | (-) | / | / |
| H-84 | female | 39 | (-) | / | / |
| H-85 | female | 56 | (-) | / | / |
| H-86 | male | 37 | (-) | / | / |
| H-87 | female | 34 | (-) | / | / |
| H-88 | male | 50 | (-) | / | / |
| H-89 | male | 75 | (-) | / | / |
| H-90 | male | 34 | (-) | / | / |
| H-91 | female | 29 | (-) | / | / |
| H-92 | female | 54 | (-) | / | / |
| H-93 | female | 37 | (-) | / | / |
| H-94 | male | 65 | (-) | / | / |
| H-95 | female | 63 | (-) | / | / |
| H-96 | male | 52 | (-) | / | / |
| H-97 | female | 55 | (-) | / | / |
| H-98 | female | 32 | (-) | / | / |

Note: Clinical Diagnose (+) indicates patient samples that were confirmed positive by both culture-based findings and clinical diagnosis of urinary system-related diseases. Pathogen species were identified via standard culture methods. The Latin binomials of all bacterial and fungal species mentioned in this study were retrieved from the List of Prokaryotic names with Standing in Nomenclature (LPSN, https://lpsn.dsmz.de/) and Index Fungorum (https://www.indexfungorum.org/) databases to ensure nomenclatural validity and consistency.

Clinical Diagnose (-) samples were collected from health checkup participants. All urinalysis indicators for these individuals were within normal reference ranges, classified as UTI-negative.

UTI: urinary tract infection; cUTI: complicated urinary tract infection; BPH: benign prostatic hyperplasia; BC: bladder cancer; CKD: chronic kidney disease; AKI: acute kidney injury.

**Table S4 Validation of the robustness and stability of the diagnostic model by 1000 independent random train-test splits**

| Accuracy | Precision | Sensitivity | F1-score | AUC |
| --- | --- | --- | --- | --- |
| 0.944 (±0.029) | 0.944 (±0.027) | 0.9420 (±0.034) | 0.941 (±0.032) | 0.991 (±0.009) |

**Table S5. Comparative evaluation of SMART against other exemplary methods for ATP and miRNA detection.**

| **Method** | **Major components (number of enzyme and probes)** | **Detection strategy** | **Pre-amplification** | **One-pot reaction (steps)** | **LOD** | **Assay time** | **Cost per test** |
| --- | --- | --- | --- | --- | --- | --- | --- |
| SMART | 0 enzyme, 8 probes (2 probes / target) | loop activated DNAzyme | No | Yes | miRNAs: femtomolar;  ATP: picomolar | 2.5 h | $0.08 |
| Luciferin-luciferase bioluminescence | 1 enzyme, 1 probe | Chemiluminescent luciferase | Yes | Yes | ATP: nanomolar | 30 min | $1.44 |
| DNA tile^[7]^ | 0 enzyme, 3 probes / target | 3PS arrays,switch and HCR | Yes | No,2 | ATP: nanomolar | 3 h | NA |
| Triple-signal paper-based sensor^[8]^ | 0 enzyme, QCT, BSA-AuNCs, TMB | chromogenic reaction | No | Yes | ATP: micromolar | 12 min | NA |
| ID-HCR^[9]^ | 0 enzyme, 4 probes / target | HCR and toehold activated DNAzyme | Yes | Yes | ATP: nanomolar | 5 h | NA |
| DNA Tetrahedra Module^[10]^ | 0 enzyme, 3probes (1 probe / target) | Hairpin | Yes | No | miRNAs: nanomolar | 11 min | NA |
| DSN-CRISPR-MB^[11]^ | 3 enzymes, 3 probes | initiator DNA and trans-cleavage of CRISPR- Cas12a | Yes | No,3 steps | miRNAs: picomolar | 3 h | NA |
| EXTRA-CRISPER^[12]^ | 3 enzymes, 3 probes | cis- and trans-cleavage of CRISPR- Cas12a | Yes, RCA | Yes | miRNAs: femtomolar | 1.5 h | $0.60 |
| [AuNC-AuNPs-IS-AgMNPs^[13]^](mailto:AuNC@AuNPs-IS-AgMNPs) | 0 enzyme, 4 probes (2 probes / target), AuNP | hierarchical SERS-magnetic assembly | Yes, IS-AgMNPs | No,3 steps | miRNAs: attomolar | 1.5 h | NA |
| LAMP-CRISPR^[14]^ | 3 enzymes, 3 probes | CRISPR-Cas12a assisted ligation initiated LMAP | Yes, LAMP | Yes | miRNAs: attomolar | 1 h | NA |
| CHA-HCR-DNAzyme^11^ | 0 enzyme, 7 probes | cascaded CHA-HCR-DNAzyme amplification | No | Yes | miRNAs: picomolar | 4 h | NA |
| CHA-DNAzyme^12^ | 0 enzyme, 4 probes | CHA and toehold activated DNAzyme | No | Yes | miRNAs: picomolar | 5 h | NA |

Note: NA: Not Available. These cost components were not explicitly reported in the original references. To ensure accuracy, no estimated values were assigned.

**References**

[1] M. E. Fornace, J. Huang, C. T. Newman, N. J. Porubsky, M. B. Pierce, N. A. Pierce, *NUPACK: Analysis and Design of Nucleic Acid Structures, Devices, and Systems*, Chemistry **2022**.

[2] J. N. Zadeh, C. D. Steenberg, J. S. Bois, B. R. Wolfe, M. B. Pierce, A. R. Khan, R. M. Dirks, N. A. Pierce, *J. Comput. Chem.* **2011**, *32*, 170.

[3] Q.-L. Zhang, L.-L. Wang, Y. Liu, J. Lin, L. Xu, *Nat. Commun.* **2021**, *12*, 4654.

[4] H. Peng, X.-F. Li, H. Zhang, X. C. Le, *Nat. Commun.* **2017**, *8*, 14378.

[5] Y. Hu, C. Li, M. Hu, Z. Zhang, R. Fu, X. Tang, T. Wu, *Small* **2023**, *19*, 2300207.

[6] H. Peng, X.-F. Li, H. Zhang, X. C. Le, *Nat. Commun.* **2017**, *8*, 14378.

[7] D. Li, Y. Peng, L. Lu, D. Chen, B. Wang, Q. Pu, Q. Zhou, X. Xiao, M. Fu, X. Wang, J. Tu, H. Pei, Q. Wu, *Chem. Eng. J.* **2024**, *499*, 156270.

[8] P. Liu, C. Shi, Y. Liu, Z. Gai, H. Tian, F. Yang, Y. Yang, *Talanta* **2025**, *292*, 127976.

[9] Y. Gao, Y. Chen, J. Shang, S. Yu, S. He, R. Cui, F. Wang, *ACS Appl. Mater. Interfaces* **2022**, *14*, 5080.

[10] Z. Zhou, Y. S. Sohn, R. Nechushtai, I. Willner, *ACS Nano* **2020**, *14*, 9021.

[11] S. Gong, J. Li, W. Pan, N. Li, B. Tang, *Anal. Chem.* **2021**, *93*, 10719.

[12] H. Yan, *Nat. Biomed. Eng.* **2023**, *7*.

[13] J. Wu, S. Li, Y. Ma, W. Zhi, T. Chen, X. Huang, C. Huang, X. Zhou, P. Zhang, Y. Zhang, G. Zheng, Z. Wang, X. Zhong, H. Cai, W. Wang, P. Sun, H. Zhou, *Biosens. Bioelectron.* **2024**, *248*, 115993.

[14] M. Zhang, H. Wang, H. Wang, F. Wang, Z. Li, *Anal. Chem.* **2021**, *93*, 7942.

[15] H. Wang, H. Wang, Q. Wu, M. Liang, X. Liu, F. Wang, *Chem. Sci.* **2019**, *10*, 9597.

[16] L. Yang, Q. Wu, Y. Chen, X. Liu, F. Wang, X. Zhou, *ACS Sens.* **2019**, *4*, 110.
